# Assessing the translational value of animal models in developing pain-targeting therapies for clinical osteoarthritis

**DOI:** 10.1038/s43856-026-01926-7

**Published:** 2026-10-08

**Authors:** Alexandra Stubelius, Cecilia Aulin, Carla Cunha, Luminita Labusca, Christos Kontogiorgis, Gina Lisignoli, Ali Mobasheri, Rosa Maria Nothnagel, Sylvia Nürnberger, Jaqueline Lourdes Rios, Nico Sollmann, Martijn Van den Bosch, Lucienne Vonk, Carmen Corciulo

**Affiliations:** 1 Division of Molecular Bioscience, Department of Life Sciences, Chalmers University of Technology, Gothenburg, Sweden; 2 Department of Medicine Solna, Division of Rheumatology, Centre for Molecular Medicine, Karolinska Institutet, Karolinska University Hospital, Stockholm, Sweden; 3 i3S – Instituto de Investigação e Inovação em Saúde, Universidade do Porto, Porto, Portugal; 4 National Institute of Research and Development in Technical Physics, Iasi, Romania; 5 Orthopedics and Trauma Clinic, County Emergency Hospital, Iasi, Romania; 6 Laboratory of Hygiene and Environmental Protection, School of Medicine, Democritus University of Thrace, Alexandroupolis, Greece; 7 IRCCS Istituto Ortopedico Rizzoli, Laboratorio di Immunoreumatologia e Rigenerazione Tissutale, Bologna, Italy; 8 Research Unit of Health Sciences and Technology, Faculty of Medicine, University of Oulu, Oulu, Finland; 9 Department of Personalized Medicine, State Research Institute Centre for Innovative Medicine, Vilnius, Lithuania; 10 Department of Joint Surgery, The First Affiliated Hospital of Sun Yat-sen University, Guangzhou, China; 11 Faculté de Médecine, Université de Liège, Liège, Belgium; 12 Department for Health Sciences, Medicine and Research, Center for Regenerative Medicine, Faculty of Health and Medicine, University for Continuing Education Krems, Krems, Austria; 13 Department of Orthopedic Surgery, Maastricht University Medical Center, Maastricht, The Netherlands; 14 AC2T Research GmbH, Wiener Neustadt, Austria; 15 Division of Trauma-Surgery, Department of Orthopedics and Trauma-Surgery, Medical University of Vienna, Vienna, Austria; 16 Ludwig Boltzmann Institute for Traumatology, The Research Center in Cooperation with AUVA Austrian Cluster for Tissue Regeneration, Vienna, Austria; 17 Division of Medical Surgery Specialities, Department of Orthopedics, University Medical Center Utrecht, Utrecht, The Netherlands; 18 Department of Diagnostic and Interventional Neuroradiology, School of Medicine and Health, TUM Klinikum Rechts der Isar, Technical University of Munich, Munich, Germany; 19 Department of Diagnostic and Interventional Radiology, University Hospital Ulm, Ulm, Germany; 20 TUM-Neuroimaging Center, TUM Klinikum Rechts der Isar, Technical University of Munich, Munich, Germany; 21 Department of Nuclear Medicine, University Hospital Ulm, Ulm, Germany; 22 Experimental Rheumatology, Department of Rheumatology, Radboud University Medical Center, Nijmegen, The Netherlands; 23 Xintela AB, Lund, Sweden; 24 Institute of Neuroscience and Physiology, Department of Pharmacology, University of Gothenburg, Gothenburg, Sweden

**Keywords:** Osteoarthritis, Clinical pharmacology

## Abstract

Osteoarthritis (OA) is a highly prevalent and disabling disease, with pain as its most debilitating symptom. Despite extensive preclinical research, no therapy has successfully translated from animal models into routine clinical use. Commonly used animal models (e.g., acute, surgically, or chemically induced OA in young, single-sex animals) often fail to reflect the chronic and multifactorial human condition, which is often seen in older, postmenopausal women with comorbidities and mixed nociceptive–neuropathic pain. This review aims to identify animal models and pain assessment methods for predicting drug efficacy in OA pain management and to examine the translational gap, focusing on pain as a primary endpoint in animal and human clinical studies. Ultimately, we emphasize the need for phenotype-specific, clinically relevant models to improve translational validity and accelerate the development of effective OA pain therapies.

## Abstract (web summary)

Stubelius et al. compare clinical trial outcomes with preclinical animal studies to assess the translational value of osteoarthritis pain models. They identify discrepancies in timing, outcome measures, and model characteristics, and propose refinements to improve predictive validity.

## Introduction

Animal models have played a fundamental role in biomedical research, providing insights into physiological and pathological mechanisms, facilitating drug discovery, and supporting toxicological evaluation. Continued refinement of these models alongside the rapid development of non-animal alternatives^1^, will significantly improve the quality and translational value of pharmacological research.

Although the number of animals used in research peaked in the mid-1980s and has since declined due to improved welfare practices, the volume of animal-based studies has continued to grow^2^. However, this expansion has not translated into proportional clinical success. While approximately 64% of treatments showing efficacy in animal models advance to clinical trials, only 7–10% demonstrate comparable benefit in humans^3,4^. This stark discrepancy highlights a persistent translational gap^5^, driven by the difficulty of modeling complex, chronic human diseases and by limitations in experimental design, data interpretation, and the anthropocentric lens through which results are often viewed.

Osteoarthritis (OA) exemplifies these challenges. Existing animal models, whether surgical, chemical, or spontaneous, can reproduce specific disease features, such as cartilage degeneration, synovial inflammation, osteophyte formation, subchondral bone alteration, and pain-like behaviors. However, they often fall short in replicating the full complexity of human OA. Pain, in particular, remains a major translational challenge, not only in OA research but across the broader biomedical field. This challenge stems from several limitations inherent to the physiology of pain, as previously described^6^.

Pain is not merely a sensory response, but a multifaceted experience influenced by psychological factors, age, sex, molecular signaling, and neuroadaptive changes over time^7^. These dimensions are difficult to capture in animal models, which often rely on reflexive or evoked responses that may not reflect the chronic, fluctuating, and subjective nature of pain in humans^8^.

There is therefore a need for improved alignment between preclinical models and clinical reality. This review compares efficacy outcomes from clinical trials and preclinical studies, focusing on pain as a primary endpoint in OA. By integrating information on study design, evaluation methods, and pain biology, we aim to identify animal models and assessment approaches with the highest predictive value for clinical efficacy, and to support the development of more translationally relevant strategies in OA research.

### Pain mechanisms in OA

OA is often initiated by joint injuries or microtrauma to cartilage or subchondral bone, which trigger ineffective repair responses and subsequent inflammation^9^. These early events can drive structural changes and physiological disruption of joint function, ultimately resulting in chronic pain and disability^10^. It is worth noting that the severity of pain in knee OA often does not correspond closely with radiographic findings^11^. Instead, synovitis and bone marrow lesions show a stronger association with pain intensity, particularly in the context of nociceptive pain^12^. Sustained inflammation can also induce aberrant nerve sprouting, while persistent peripheral input can drive peripheral sensitization. Collectively, these processes contribute to the emergence of “neuropathic-like pain” symptoms^13^. However, it is estimated that only 23% of individuals living with OA exhibit neuropathic pain features^14^, consistent with the International Association for the Study of Pain definition of neuropathic pain as “pain caused by a lesion or disease of the somatosensory nervous system”^15^.

Pain sensitization refers to an increased responsiveness of the nervous system whereby pain signals are amplified beyond what would be expected from the degree of joint damage in OA. It encompasses both peripheral sensitization, characterized by heightened excitability of nociceptors within the affected joint, and central sensitization, in which spinal and supraspinal circuits magnify pain perception. As a result, individuals with OA may experience pain that is more intense, widespread, and persistent than expected based on structural pathology alone^16^.

Comorbidities further complicate the clinical picture: OA is associated with up to 30 coexisting conditions, including depression, anxiety, and diabetes, all of which can substantially influence pain perception^17^. Anxiety, for example, can heighten pain sensitivity and reduce tolerance to noxious stimuli through activation of the amygdala and other limbic structures, which modulate descending pain pathways, leading to enhanced nociceptive processing^18^. Diabetes can alter afferent sensory input through peripheral neuropathy, manifesting as allodynia and hyperalgesia, or sensory loss, while central involvement (diabetic encephalopathy) may affect cortical and subcortical processing of nociceptive, affective and cognitive pain dimensions^19^.

Ageing introduces additional complexity, and age-related changes in myelinated and unmyelinated fibres, along with altered nociceptor sensitization, can modify peripheral afferent input, while changes in brain connectivity and reduced endogenous pain inhibition may affect central pain processing^20^. Thus, in older OA cohorts, pain severity may reflect a composite of joint pathology, peripheral sensory alteration, central sensitization, and cognitive modulation.

### OA animal models and pain assessment in laboratory animals

OA animal models can be broadly classified as either spontaneous models, where disease develops due to aging or genetic predisposition, or induced models, where OA-like pathology is triggered through mechanical, surgical, chemical, or genetic interventions. These models have been established in at least 18 different species, ranging from small vertebrates such as zebrafish to larger mammals, including non-human primates^21^. Each model captures distinct aspects of joint pathology, biomechanic, or immune system involvement.

Surgical models, such as destabilization of the medial meniscus (DMM) in mice or anterior cruciate ligament transection (ACLT) in rats and rabbits, resemble features of post-traumatic OA and provide rapid and reproducible joint degeneration suitable for mechanistic or therapeutic studies. Similarly, medial meniscus resection (MMX) induces a comparable OA phenotype, although it is characterized by pronounced bone degeneration and more rapid disease progression^21^. In surgical models of osteoarthritis, pain progression is typically characterized by an initial post-surgical phase lasting 1–3 weeks, during which pain is largely attributable to surgical trauma and remains responsive to anti-inflammatory treatment. As inflammation resolves, a relatively quiescent phase generally follows until approximately 6–8 weeks post-induction. Thereafter, chronic pain develops. However, both the duration of the quiescent phase and the onset of chronic pain vary considerably across studies, depending on the model used, the pain assessment methods employed, and animal-related factors such as sex and age. In particular, rodents younger than 12 weeks often display an earlier onset of pain behaviors^22–24^.

The monoiodoacetate (MIA) model induces biphasic pathology, with early immune activation (days 0–7), followed by OA related pain (days 14–28). Alterations of the cartilage surface are observed by day 14 after induction, with cartilage fissures evident by day 28^25,26^. Pain-related behaviors, including mechanical allodynia, tipically appears 5–10 days after MIA injection in mice (depending on the MIA dose), while weight-bearing deficits can be detected as early as day 3 post-injection^27^.

Pain in OA models is assessed using a range of experimental paradigms. Some of these assays measure evoked pain, which is reported in approximately 15–50% of patients with chronic pain^28^. Among these, the von Frey test and the knee bending test assess mechanical allodynia, the Randall–Selitto paw pressure test evaluates mechanical hyperalgesia, the acetone test assesses thermal allodynia, and thermal hyperalgesia can be measured using the Hargreaves apparatus (radiant heat) or hot and cold plate tests. In contrast, clinically symptomatic human OA is defined by patient-reported pain, which may include spontaneous/rest pain as well as movement- or weight-bearing–evoked pain. In animal models, spontaneous pain is commonly assessed using static or dynamic weight bearing (SWB and DWB, respectively) and gait analysis, which are among the most widely applied methods in OA research^29^. Ambulatory-evoked pain assessment, analysis of spontaneous locomotor activity, wheel running, and burrowing behavior tests are used as indirect measures of spontaneous pain^30^.

### Pain assessment in clinical subjects

In clinical studies, pain intensity is most typically assessed using self-reported questionnaires based on numerical or visual scales. The most common tools include the Visual Analogue Scale (VAS) and the Numeric Rating Scale (NRS), which quantify pain intensity on a continuous or numerical scale, respectively^31^. The Patient Global Assessment (PGA) is instead a broad numerical assessment of the disease activity^32^. Comprehensive questionnaires such as the Western Ontario and McMaster Universities Osteoarthritis Index (WOMAC) and the Knee Injury and Osteoarthritis Outcome Score (KOOS) provide multidimensional evaluations of pain, stiffness, and physical function^33^. Additionally, functional performance tests provide objective insights into how pain impacts daily mobility and endurance. For a more mechanistic assessment, quantitative sensory testing (QST) can be used to evaluate pressure and thermal pain thresholds, providing information on peripheral and central sensitization^34,35^.

## Data collection

**Fig. 1:**
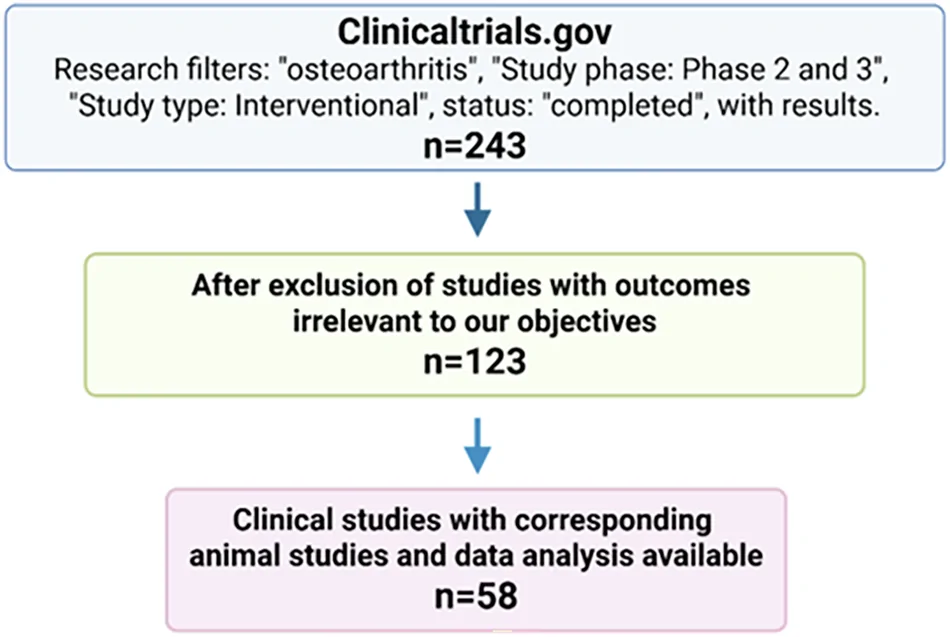
Workflow for the selection of clinical and preclinical studies. Clinical trials were identified through a search of ClinicalTrials.gov. Following the exclusion of studies outside the scope of this review, trials were further screened to identify compounds with publicly available clinical data and corresponding preclinical studies assessing their efficacy on pain-related outcomes in animal models.

We identified interventional phase 2 and phase 3 studies on ClinicalTrials.gov with published results up to May 2025 that evaluated pharmacological agents using pain as the primary endpoint. Where available, external references were included to provide additional context. Studies were excluded if they lacked data analysis or reported outcomes not aligned to our objectives, including those focused solely on structural modification, inflammation, safety, use of devices, comparison between drugs, or conditions associated with OA or joint replacement surgery (without directly addressing OA pain). Studies evaluating viscosupplementation products, herbal-derived compounds, or homeopathic preparations were excluded due to the absence of a clearly defined mechanism targeting pain. Only studies for which publicly available data from OA animal models existed, whether generated in direct support of the clinical trial or independently, were included in the analysis (Fig. 1, Supplementary Table 1).

Studies on laboratory animals were identified through PubMed using the search strategy: ((drug name[MeSH Terms] OR drug name[Title/Abstract]) AND (osteoarthritis[MeSH Terms] OR osteoarthritis[Title/Abstract] OR osteoarthrit*[Title/Abstract])) AND (pain[MeSH Terms] OR pain[Title/Abstract] OR analgesia[Title/Abstract]). The search was restricted to English-language publications and filtered for “other animals”. Studies were excluded if they lacked a control group, if concerns regarding data integrity or reporting transparency had been raised by journal editors, if pain outcomes were not directly evaluated, or if they referred to veterinary reports of spontaneous OA in companion animals.

### Summary of the characteristics of the participants in the clinical studies

Most clinical studies required individuals with OA to be at least 40 years old, although in some cases the minimum age was 18 years. The average age of participants across studies (123 studies) was 61.4 ± 8.7 years (weighted values). Both men and women were represented, with women comprising a weighted average of 66% of the study populations. Body mass index (BMI) greater than 39–40 kg/m² was an exclusion criterion in some studies. Most studies required a duration of knee pain of at least 3 to 6 months as an inclusion criterion. Some studies also reported baseline pain levels corresponding to mild-to-severe pain. An overview of the characteristics of the studies’ participants is summarized in Supplementary Table 2.

In the following section, we summarize data from human clinical trials and animal studies across different pharmacological classes of compounds.

## Case studies

### Non-steroidal anti-inflammatory drugs

NSAIDs remain among the most widely used pharmacological treatments for OA because of their ability to inhibit cyclooxygenase (COX) enzymes and reduce inflammation and pain. Current guidelines from the Osteoarthritis Research Society International (OARSI) recommend NSAIDs as first-line therapy for OA patients without major comorbidities^36^.

Diclofenac is one of the most extensively studied NSAIDs in both clinical and preclinical OA research. In a randomized placebo-controlled trial (NCT01461369), oral diclofenac capsules (35 mg three times/day) significantly improved WOMAC pain, stiffness, and physical function scores in knee and hip OA patients after 12 weeks of treatment^37^. Conversely, neither topical administration four times daily for 4 weeks (NCT05007808) nor intra-articular (i.a.) diclofenac etalhyaluronate administered every 4 weeks for 12 weeks (NCT03209362) demonstrated significant efficacy compared with the placebo-treated group. In preclinical OA models, particularly MIA-induced OA in rats, diclofenac consistently reduced mechanical allodynia, thermal hypersensitivity, weight-bearing asymmetry, and grip force deficits following oral, intraperitoneal (i.p.), subcutaneous (s.c.), or topical administration. However, efficacy appeared greater during early inflammatory phases, with reduced benefit in later chronic stages and in gait-related assessments^25,38–51^. An exception was diclofenac etalhyaluronate, which showed sustained efficacy for up to 8 weeks after a single injection administered 28 days following OA induction^52^.

Low-dose SoluMatrix Meloxicam, a preferential COX-2 inhibitor, also demonstrated clinical efficacy (5–10 mg once daily). Placebo-controlled trials in knee and hip OA patients reported significant improvements in WOMAC pain and function scores over 12 weeks (NCT01787188)^53^. In animal studies, meloxicam reduced mechanical hypersensitivity and thermal hyperalgesia in MIA and ACLT models, although effects on spontaneous pain and gait behaviors were less consistent^54–59^.

Other selective COX-2 inhibitors, such as etoricoxib and celecoxib, also showed good translational concordance. In randomized clinical studies (NCT01554163, NCT00092768, NCT00092781, NCT01980940), etoricoxib and celecoxib similarly improved WOMAC outcomes in knee OA patients^60,61^. Correspondingly, preclinical studies demonstrated reductions in mechanical allodynia, thermal hypersensitivity, and weight-bearing deficits across MIA, ACLT, DMM, and mechanical loading models^62–81^.

In contrast with previously described NSAIDs, paracetamol showed limited efficacy. Clinically, sustained-release paracetamol failed to improve WOMAC pain scores versus placebo in knee OA patients (NCT02311881)^82^. Preclinical studies on MIA rat model reported only transient reductions in pain-related behaviors, with little efficacy during chronic disease stages^38,39^.

Details of the studies for the evaluation of the NSAIDs efficacy on OA pain are described in Table 1.

**Table 1:** Selected clinical and preclinical studies evaluating the efficacy of NSAIDs, including study design, treatment characteristics, and pain-related outcomes

	Affected joint/OA model	Dose	Administration route and frequency	Timing of treatment initiation	Outcome for pain assessment	Timepoint of efficacy assessment	Outcome: efficacy in pain reduction	References
Diclofenac	Knee, hip	35 mg	p.o./twice daily	≥6 months	WOMAC	After 12 weeks of treatment	Efficacy superior to placebo	NCT01461369^37^
	Knee	4 gr	Topical/four time daily	≥6 months	WOMAC pain	After 4 weeks of treatment	Efficacy not superior to placebo	NCT05007808 PNA
	Wistar rats/MIA	30 mg/kg	s.c./before test	3 days post OA induction	Mechanical allodynia	3 days after OA induction	Effective at day 3 of OA induction; no effect at later time points	^38^
	Sprague Dawley rats/MIA	3 mg/kg	p.o./daily	7 days post OA induction	SWB	10, 14, 21 and 28 days after OA induction	Effective on day 10 of OA induction; no effect at later time points	^25^
	Sprague Dawley rats/MIA	10–100 µmol/kg	p.o./20 min before first test	20 days post OA induction	HGS	20–60 min after dose	Efficacy superior to the vehicle	^39^
	Cynomolgus macaques/MNX	10 mg/kg	p.o./daily	7 days post OA induction	SWB, mechanical hyperalgesia	3 h after the dose	Efficacy not superior to the vehicle	^40^
	C57BL/6J/MIA	10 mg/kg	i.p./daily	14 days post OA induction	Mechanical allodynia	7, 14, 21, 28 days after OA induction	Efficacy superior to the vehicle from day 14	^41^
	Sprague Dawley rats/MIA	10 mg/kg	p.o./daily	7 days before OA induction	Mechanical hyperalgesia	Weekly for 5 weeks	Efficacy superior to the vehicle	^42^
	C57BL/6J/MIA	10 mg/kg	p.o./daily	14 days post OA induction	SWB, Mechanical allodynia	Weekly for 5 weeks	Efficacy superior to the vehicle at week 4	^43^
	Sprague Dawley rats/MIA	10 mg/kg	p.o./twice	3 and 7 days post OA induction	SWB	2, 4, 6 h post dose	Efficacy superior to the vehicle	^44^
	Wistar rats/MIA	1%/1 ml	Topical/before test	1, 4, 7 and 14 days post OA induction	Mechanical and thermal allodynia, thermal hyperalgesia, and spontaneous allodynia	1, 4, 7 and 14 days after OA induction	Efficacy superior to the vehicle	^45^
	Wistar rats/MIA	1%/0.4 gr	Topical/daily	1 days post OA induction	Mechanical and thermal allodynia, thermal hyperalgesia, and SPB.	1–21 days after OA induction	Efficacy superior to the vehicle	^46^
	Sprague Dawley rats/MIA	2 mg/kg	p.o./daily	0 day post OA induction	Mechanical allodynia	Up to 4 weeks of treatment	Efficacy superior to the OA group without treatment	^47^
	Sprague Dawley rats/MIA	3–30 mg/kg	p.o./2 h before testing	21 days post OA induction	SWB	21 and 28 days after OA induction	Efficacy not superior to the vehicle group	^48^
	Wistar rats/MIA	30 mg/kg	p.o./2 h before testing	3 days post OA induction	Gait analysis, mechanical hyperalgesia	3 and 20 days after OA induction	Efficacy relative to the contralateral joint at day 3. No effect was detected in gait analysis at day 20	^49^
	Hartley Guinea pig/MIA shoulder	10 mg/kg	p.o./6 h before test	7 days post OA induction	SWB	7 days after OA induction	Efficacy superior to the vehicle	^50^
	Sprague Dawley rats/MIA	3, 10, 30 mg/kg	p.o./single dose	21 days post OA induction	SWB, gait analysis	2 h before tests	Efficacy not superior to the vehicle	^51^
Diclofenac Etalhyaluronate	Knee	30 mg	i.a./every 4 weeks	n.a.	WOMAC pain	After 12 weeks	Efficacy not superior to the vehicle	NCT03209362
	Sprague Dawley rats/MIA	0.5 mg	i.a./once	28 days after OA induction	Mechanical hyperalgesia	Weekly for 8 weeks	Efficacy superior to the vehicle	^52^
Meloxicam	Knee, hip	5 and 10 mg	p.o./daily	n.a.	WOMAC	After 12 weeks of treatment	Efficacy superior to placebo at the higher dose	NCT01787188^53^
	*Rattus norvegicus*/MIA	1, 3, 10 mg/kg	p.o./daily	21 days post OA induction	Thermal allodynia	Up to 4 weeks of treatment	Efficacy superior to the vehicle at the higher dose	^54^
	Sprague Dawley rats/MIA	20 mg/kg	i.m./daily	3 days post OA induction	Mechanical and thermal allodynia, HGS, SWB	3, 7, 14 days after OA induction	Efficacy superior to the vehicle	^55^
	Sprague Dawley rats/MIA	1 mg/kg	p.o./daily	3 days post OA induction	Mechanical and thermal allodynia, SWB	3, 7, 14 days after OA induction	Efficacy superior to the saline No effect on the gait analysis	^56^
	C57Bl/6J	5 mg/kg	i.p./90 min before test	10 days post OA induction	Mechanical allodynia, SPB	10 days after OA induction	Efficacy superior to the saline	^57^
	Sprague Dawley rats/MIA	1–10 mg/kg	i.p./single dose	10 days post OA induction	Mechanical allodynia	10–42 days after OA induction	Efficacy not superior to the vehicle group	^58^
	Wistar rats/MIA	6 mg/kg	i.p./daily	4 days post OA induction	Modified SWB	4–7 days after OA induction	Efficacy superior to the untreated group	^59^
Etoricoxib	Knee	30 mg	p.o./daily	n.a.	WOMAC pain subscale	After 12 and 26 weeks of treatment	Efficacy superior to placebo	NCT00092768^61^
	Knee, hip	30 mg	p.o./daily	n.a.	WOMAC pain subscale	After 12 and 26 weeks of treatment	Efficacy superior to placebo	NCT00092781^61^
	Knee	75, 150, 163 mg	Topical	≥ 6 months	WOMAC pain subscale	After 2 weeks of treatment	Efficacy not superior to placebo	NCT01980940 PNA
	Wistar rats/ACL rupture	6.7 and 33.3 mg/kg	p.o./3 times per week	8 weeks post OA induction	Mechanical allodynia, SWB	Up to 21 weeks post-surgery	Efficacy superior to the placebo from 12 weeks post-surgery	^77^
	Wistar rats/ACL rupture	0.01–20 µg/rat (i.t.) or 1.5, 3, 6 mg/kg (p.o.)	i.t. or p.o./30 min before test	2 and 16 weeks 3 post OA induction	Mechanical allodynia	30–180 min after dose	Efficacy superior to the vehicle	^78^
	Sprague Dawley rats/MIA	10 mg/kg	p.o./daily	0 day post OA induction	Mechanical hyperalgesia, thermal allodynia, SPB	0, 7, 14 days after OA induction	Efficacy superior to the vehicle	^79^
	Wistar rats/MIA	10 mg/kg	p.o./daily	0 day post OA induction	Mechanical hyperalgesia, thermal allodynia, SPB	0, 7, 14 days after OA induction	Efficacy superior to the vehicle for all the tests but the SPB	^80^
	Wistar rats/MIA	10 mg/kg	p.o./daily	0 day post OA induction	Mechanical hyperalgesia, thermal allodynia, SPB	0, 7, 14, 21 days after OA induction	Efficacy superior to the vehicle	^81^
Celecoxib	Knee	200 mg	p.o./daily	≥6 months	WOMAC pain subscale	After 12 weeks of treatment	No difference in efficacy compared with Etoricoxib	NCT01554163^60^
	C57BL/6J/DMM	1, 5, 25 or 125 mg/kg	p.o./daily	0 day post OA induction	Mechanical and hyperalgesia, SPB	4 weeks after OA induction	Efficacy superior to the saline	^62^
	C57BL/6J/DMM	10 mg/kg	i.g./every 2 days	7 days post OA induction	Thermal allodynia, SPB	3–56 days after OA induction	Efficacy superior to the untreated group	^63^
	Sprague Dawley rats/MIA	3, 10, 30 mg/kg (acute) or 30 mg/kg (chronic)	p.o./daily for 30 days or 1 h before tests	21 days post OA induction	SWB	1 h or 10 days after treatment	Efficacy superior to the saline	^64^
	Wistar rats/ACLT	0.01–20 µg/rat (i.t.) or 1.5, 3, 6 mg/kg (p.o.)	i.t. or p.o./30 min before test	2 and 16 weeks post OA induction	Mechanical allodynia	30–180 min after treatment	Efficacy superior to the vehicle	^78^
	Sprague Dawley rats/MIA	10 mg/kg	p.o.	7 and 14 days post OA induction	SWB	28 days after OA induction	Efficacy superior to the vehicle	^65^
	Sprague Dawley rats/MIA	3 mg/kg	NA	0 day post OA induction	SWB	1–4 weeks after treatment	Efficacy superior to the vehicle	^66^
	Sprague Dawley rats/MIA	50 mg/kg	p.o./5 days per weeks	0 day post OA induction	SWB	After 6 weeks of treatment	Efficacy superior to the vehicle	^67^
	Sprague Dawley rats/MIA	10–100 umol/kg (acute) or 10–30 umol/kg (chronic)	p.o/20 min before the first test or twice day for 14 days	20 (acute) or 7 (chronic) days post OA induction	HGS	20–60 min after dose (acute) or 14 days of treatment	Efficacy superior to the vehicle	^39^
	Sprague Dawley rats/MIA	100 mg/kg	p.o./daily for 14 days	n.a.	SWB	4–14 days after OA induction	Efficacy superior to the vehicle	^68^
	C57BL/6J/ACLT	10 mg/kg	p.o./daily for 28 days	4 weeks post OA induction	Mechanical allodynia, SPB	After 28 days of treatment	Efficacy superior to the vehicle in allodynia measurement. No effect on the SPB	^69^
	Sprague Dawley rats/MIA	3 mg/kg	p.o./daily for 4 weeks	7 days post OA induction	SWB	Up to 35 days of treatment	Efficacy superior to the vehicle	^70^
	Wistar Han rats/bilateral MIA	3–30 mg/kg	p.o./single dose	3 days post OA induction	SPB	2 h after treatment	Efficacy superior to the vehicle	^71^
	Wistar rats/MIA	1–10 mg/kg	p.o./before test	14, 21, 28 days post OA induction	SWB	1, 2, 4 h after treatment	Efficacy superior to the vehicle	^72^
	C57BL/6J/Mechanical loading	40 mg/kg	p.o.	1 day before OA induction	Gait analysis, Mechanical allodynia	After 3–7 days of treatment	Efficacy superior to the vehicle-treated group	^73^
	Sprague Dawley rats/MIA	3 mg/kg	p.o./single dose	14 days post OA induction	Mechanical hyperalgesia	3 and 24 h after treatment	Efficacy not superior to the vehicle	^74^
	Wistar rats/MIA	3–30 mg/kg	n.a.	1 day post OA induction	Mechanical allodynia	30–240 min after treatment	Efficacy superior to the vehicle	^75^
	C57BL/6J/MIA	20 mg/kg	p.o. gavage/daily	5 days post OA induction	SWB	Every 3 days	Efficacy superior to the vehicle	^76^
Paracetamol	Knee	1000 or 665 mg	p.o./twice daily	≥3 months	WOMAC pain subscale	After 12 weeks of treatment	Efficacy not superior to placebo	NCT02311881^82^
	Wistar rat/MIA	300 mg/kg	p.o./1 h before test	3 days post OA induction	Mechanical allodynia, SWB	One hour after treatment and 14 and 28 days after OA induction	SWB improves 1 h after treatment; no efficacy on allodynia at 14 and 28 days after OA induction	^38^
	Sprague Dawley rats/MIA	30–300 µmol/kg	p.o./20 min before first test	20 days post OA induction	HGS	20–60 min after treatment	Efficacy superior to the vehicle	^39^

*ACLT* anterior cruciate ligament transection, *HGS* hindlimb grip strength, *i.m.* intramuscular, *i.t.* intrathecal, *MIA* monosodium iodoacetate, *MNX* medial meniscal transection, *n.a.* not available, *PNA* publication not available, *p.o.* per oral, *s.c.* subcutaneous, *SPB* spontaneous pain behavior, *SWB* static weight bearing, *WOMAC* Western Ontario and McMaster Universities Osteoarthritis Index.

Overall, NSAIDs demonstrated good concordance in efficacy across studies, independently of dose and route of administration, with efficacy observed in nearly all cases except for paracetamol. However, an important discrepancy concerns the timing of treatment initiation: in clinical trials NSAID therapy is generally started after at least 3–6 months of symptoms, whereas in animal studies, treatment is often initiated at the time of OA induction or during the early stages of disease development. Notably, 36 of the 45 preclinical studies analyzed were performed using the MIA model. Lack of efficacy was mainly observed when NSAIDs were administered at later stages in the MIA model or in the single primate study, suggesting that both species differences and treatment timing may influence outcomes. These findings indicate that NSAIDs may require chronic administration initiated during the early phases of disease progression to demonstrate efficacy in preclinical OA models.

### Extended-release and combination corticosteroids

Corticosteroids are widely used anti-inflammatory agents for OA management. Because of their rapid intra-articular clearance, extended-release formulations have been developed to prolong analgesic efficacy while reducing injection frequency and systemic adverse effects. Among these, triamcinolone acetonide extended release (TA-ER, FX006), a poly(lactic-co-glycolic acid)-based microsphere formulation, has shown promising clinical results. In Phase IIb and Phase III clinical trials (NCT02116972 and NCT02357459), single i.a. injections of TA-ER (16–32 mg or 40 mg) significantly improved pain outcomes in knee OA patients, with reductions in weekly NRS scores maintained up to 12 weeks^83^. Preclinical studies generally support the analgesic efficacy of intra-articular corticosteroids across multiple OA models. Most investigations were performed in MIA-induced OA in rats, where triamcinolone acetonide improved gait, mechanical allodynia, paw pressure, balance, and locomotor activity following a single intra-articular administration. These effects were observed in both Wistar rat and Sprague-Dawley rat models, with some studies reporting sustained benefit for up to 60 days^84–86^. However, efficacy was less consistent in surgically induced OA models such as meniscal transection, where improvements in mechanical allodynia and weight-bearing deficits were not observed^87,88^. By contrast, positive effects were reported in rabbits and C57BL/6 mouse models^89,90^. A summary of studies evaluating triamcinolone efficacy in OA pain is provided in Table 2.

**Table 2:** Selected clinical and preclinical studies evaluating the efficacy of corticosteroids, including study design, treatment characteristics, and pain-related outcomes

Drug	Affected joint/ OA model	Dose	Administration route and frequency	Timing of treatment initiation	Outcome for pain assessment	Timepoint of efficacy assessment	Outcome: efficacy in pain reduction	References
Triamcinolone	Knee	16–32 mg	i.a./single dose	≥6 months	NRS	12 weeks after treatment	Efficacy superior to placebo	NCT02116972^83^
	Knee	40 mg	i.a./single dose	≥6 months	NRS	12 weeks after treatment	Efficacy superior to placebo	NCT02357459^83^
	Wistar rat/MIA	2 mg/kg	i.a./single dose	4 weeks post OA induction	Gait analysis	4 weeks after treatment	Improvement vs baseline	^84^
	Sprague Dawley rat/MIA	40 mg/ml	i.a./single dose	7 days post OA induction	Mechanical hyperalgesia, gait analysis	7, 14, 21 days after treatment	No improvement in mechanical hyperalgesia; improvement in gait analysis compared toonehicle at day 14	^85^
	Sprague Dawley rat/MNX	200 µg/µl	i.a./single dose	14 days post OA induction	Mechanical allodynia, SWB	Up to day 28 after treatment	No improvement compared to the vehicle	^87^
	Rabbit/MIA	2 mg/week	i.a./weekly	n.a.	Wire‑walking test, thermal allodynia	1, 3, 7, 14, 21, 28 days after first dose	Improvement compared to saline	^89^
	C57BL/6 mouse/partial MNX	40 µg/mouse	i.a./single dose	6 weeks post OA induction	Mechanical allodynia	1 and 3 days after treatment	Improvement compared to PBS	^90^
	Sprague Dawley rat/MIA	100 ug	i.a./single dose	7 days post OA induction	Mechanical allodynia, SWB, SPB	14, 30, 60 days after treatment	Improvement compared to vehicle at 14 and 30 days after dose. Up to 60 days for the mechanical allodynia.	^86^
	Sprague Dawley rat/MIA or MNX	1 mg/rat	i.a./single dose	14 days after OA induction	SWB, Mechanical allodynia	1 and 7 days after treatment	Efficacy superior to the vehicle only for the MNX model, not on the MIA model	^88^

*MIA* monosodium iodoacetate *MNX* medial meniscal transection *n.a.* not available *NRS* numerical rating scale *PBS* phosphate-buffered saline.

Overall, the corticosteroids show good agreement between clinical and preclinical findings, with consistent analgesic effects independent of species and OA model. However, discrepancies arise regarding the treatment timing: clinical studies typically include patients with established disease (at least 6 months of symptoms), whereas treatment in animal models is often initiated at earlier stages of disease. In the MIA model, corticosteroids were ineffective when administered 14 days after OA induction, corresponding to the chronic phase, but showed benefit when given at earlier stages. Improvements were observed in both evoked and spontaneous pain outcomes, although effects on mechanical hyperalgesia in the MIA model were limited.

### Opioids

Opioids have been investigated for OA pain management, particularly in patients with moderate-to-severe symptoms insufficiently controlled by first-line therapies. These agents primarily act through central opioid receptors, although some compounds also modulate monoaminergic pathways. Tramadol, a synthetic opioid with dual μ-opioid receptor agonism and serotonin/norepinephrine reuptake inhibition, demonstrated clinical efficacy in knee OA patients. In randomized trials evaluating tramadol Contramid formulations (NCT00852917 and NCT00833794), oral administration significantly improved pain and physical function compared with placebo, particularly at doses of 200–300 mg/day, as assessed by PGRP and WOMAC scores after 12 weeks of treatment^91^.

Preclinical studies largely supported these findings. Most investigations were conducted in MIA-induced OA in Sprague Dawley rats. Acute i.p. administration (3–100 mg/kg) reduced mechanical hypersensitivity and weight-bearing deficits, with higher doses producing analgesic effects lasting up to 2–3 h^92^. Additional studies reported dose-dependent improvements in hind limb grip force, gait performance, and functional impairment after repeated administration^39,93^. Oral tramadol similarly improved weight distribution in chronic OA models^48,51^, while in the mouse model of partial meniscectomy, a single dose of tramadol showed an effect on the mechanical allodynia but not on the thermal allodynia and mechanical hyperalgesia^94^.

Tapentadol, combining μ-opioid receptor agonism with norepinephrine reuptake inhibition, also demonstrated efficacy in OA. Clinical trials (NCT00421928, NCT00486811, NCT01124604, NCT00487435, NCT00982280, and NCT00983073) showed significant improvements in NRS pain scores at doses of 100–250 mg/day over treatment periods up to 52 weeks^95–98^. Preclinical studies demonstrated reductions in thermal allodynia in MIA-induced OA in Wistar rats^99^.

In contrast, findings for buprenorphine have been less consistent. Buccal film administration demonstrated significant improvements in NRS pain scores over a 48-week treatment period (NCT01755546)^100^, whereas transdermal administration for 12 weeks failed to show superiority over placebo (NCT03335314). In preclinical studies, intraperitoneal administration of buprenorphine in rats 6 months after ACLT produced significant analgesic effects 30 min after dosing; however, these effects were not sustained at 24 h post-administration^101^.

Details of the studies evaluating the efficacy of opioids on OA pain are summarized in Table 3.

**Table 3:** Selected clinical and preclinical studies evaluating the efficacy of opioids, including study design, treatment characteristics, and pain-related outcomes

Drug	OA model/affected joint	Dose	Administration route	Timing of treatment initiation	Outcome for pain assessment	Timepoint of efficacy assessment	Outcome: efficacy in pain reduction	References
Tramadol	Knee	100–400 mg/day	Oral/once or twice daily	<1 year	WOMAC pain subscale	After 12 weeks of treatment	Efficacy compared to the baseline, no difference between once or twice daily	NCT00950651
	Knee	100, 200 or 300 mg/day	Oral/daily	<5 years	PGA, WOMAC pain subscale	After 12 weeks of treatment	Efficacy superior to placebo (200 and 300 mg doses)	NCT00832416
	Knee	100, 200 or 300 mg/day	Oral/daily	<5 years	PGA, WOMAC -pain and physical function subscales	After 12 weeks of treatment	Efficacy superior to placebo (200 and 300 mg doses)	NCT00852917 PNA
	Knee	200 or 300 mg/day	Oral/daily	<5 years	Pain Intensity score	After 12 weeks of treatment	Efficacy superior to placebo	NCT00833794^91^
	Sprague Dawley rats/hip MIA	10 mg/kg	i.p./daily	2 weeks post OA induction	Mechanical allodynia	After 4 weeks of treatment	Efficacy superior to the vehicle	^93^
	C57BL/6 mice/partial MNX	50 mg/kg	s.c./1 h before the test	6 weeks post OA induction	SWB, mechanical and cold allodynia and hyperalgesia, SPB	After 1 h of treatment	Efficacy superior to vehicle on mechanical allodynia; no effects on paw pressure or cold plate test	^94^
	Sprague Dawley rats/MIA	10–100 umol/kg	i.p./20 min before first test	20 days post OA induction	HGS	After 20–60 min of treatment	Efficacy superior to the vehicle	^39^
	Sprague Dawley rats/MIA	3, 10, 30, 100 mg/kg	p.o./single dose	3 weeks post OA induction	SWB, gait analysis	After 2 h of treatment	Efficacy superior to the vehicle-treated group for the 10–100 mg/kg doses and the 100 mg/kg for the gait analysis	^51^
	Sprague Dawley rats/MIA	10, 30, 100 mg/kg	i.p./daily for 20 days	0 day post OA induction	HGS	After 1 h of treatment	Efficacy superior to the vehicle at the two higher doses	^39^
	Wistar rats/MIA	10 mg/kg	p.o./Oral, single dose	5 days post OA induction	SWB	After 1, 3, 6 and 24 h of treatment	Efficacy superior to untreated MIA group	^161^
	Sprague Dawley rats/MIA	3, 10, 30, 100 mg/kg	i.p./single dose	14 days post OA induction	Mechanical allodynia, SWB	Up to 3 h after treatment	Improvement compared to the baseline up to 2 h after dosing (with doses ≤10 mg/kg)	^92^
	Sprague Dawley rats/MIA	3–30 mg/kg	p.o./2 h before testing	21 days post OA induction	SWB	After 21 and 28 days of OA induction	Efficacy superior to the vehicle at doses 10 and 30 mg/kg	^48^
Tapentadol	Knee	50–500 mg/day	Oral/twice daily	>3 months	NRS	Up to 12 weeks	Efficacy superior to placebo	NCT00983073^95^
	Knee	100–250 mg	Oral/twice daily	>3 months	NRS	Up to 12 weeks of maintenance dose	Efficacy superior to placebo	NCT00421928^97^
	Knee	100–250 mg	Oral/twice daily	Not available	NRS	52 weeks of maintenance dose	Efficacy superior to placebo	NCT00487435^97^
	Knee	100–250 mg	Oral/twice daily	>3 months	NRS	15 weeks	Efficacy superior to placebo	NCT00486811^98^
	Knee	25–250 mg	Oral/twice daily	>12 weeks	NRS	12 weeks of treatment	Efficacy superior to placebo	NCT01124604 PNA
	Wistar rat/MIA	10 mg/kg	p.o./single dose	4 weeks post OA induction	Thermal allodynia	After 4 weeks of OA induction	Efficacy superior to saline	^99^
Buprenorphine	Knee	300–900 mg	Buccal film/twice daily	≥3 months	NRS	After 48 weeks of treatment	Efficacy compared to the baseline	NCT01755546^100^
	Knee	n.a.	Transdermal/continuous	≥12 months	Average pain over the last 24 h	After 12 weeks of treatment	Efficacy not superior to the placebo	NCT00531427 PNA
	Sprague Dawley rats/ACLT	0.025 mg/kg	i.p./single dose	3 and 6 months post OA induction	Gait analysis	After 30 min and 24 h of treatment	Inconclusive, efficacy compared to pre-treatment only at 30 min 6 months after OA induction	^101^

*ACLT* anterior cruciate ligament transection, *HGS* hindlimb grip strength, *i.p.* intraperitoneal, *MIA* monosodium iodoacetate, *MNX* medial meniscal transection, *n.a.* not available, *NRS* numerical rating scale, *PGA* Patient Global Assessment, *p.o.* per oral, *s.c.* subcutaneous, *SPB* spontaneous pain behavior, *SWB* static weight bearing, *WOMAC* Western Ontario and McMaster Universities Osteoarthritis Index.

Overall, preclinical and clinical studies evaluating the efficacy of opioids for OA pain showed generally good agreement, with efficacy reported in both settings independently of dose, route of administration, and timing of treatment initiation. A notable discrepancy concerns treatment frequency, which is typically chronic (daily) in patients but often acute in animal studies. Of the 11 preclinical studies identified, 9 used the MIA model, while one employed the MNX model, in which no efficacy against allodynia was observed.

### Antidepressants and anticonvulsants

Antidepressants and anticonvulsants are increasingly used for OA pain management because of their ability to modulate central sensitization and pain processing pathways. Current recommendations from OARSI support the use of anti-depressant duloxetine as a treatment for patients with knee OA and widespread pain^102^. Duloxetine, a selective serotonin and norepinephrine reuptake inhibitor, demonstrated consistent efficacy across several clinical studies. Oral administration at 60–120 mg/day significantly improved pain and physical function over treatment periods ranging from 8 to 52 weeks. Randomized placebo-controlled trials (NCT00433290, NCT00408421, NCT02335346, NCT01931475) reported reductions in Brief Pain Inventory scores, WOMAC pain and function scores, and numerical pain ratings, together with improvements in patient global impression and quality-of-life measures^103–106^. Duloxetine also showed efficacy when combined with NSAIDs, and long-term studies confirmed sustained analgesic benefits for up to 10 weeks of treatment^104^.

Preclinical findings generally supported these clinical observations. Most studies used MIA-induced OA in Sprague-Dawley rats or Wistar rats. Duloxetine consistently reduced mechanical hypersensitivity, tactile allodynia, and functional impairments following oral, intraperitoneal, or subcutaneous administration. Improvements were observed in von Frey testing, hind limb grip force, Randall–Selitto thresholds, and weight-bearing asymmetry^39,51,74,107^. Repeated treatment also improved spontaneous and affective pain-related behaviors, including burrowing activity^108^.

In contrast, the anticonvulsant sodium channel blocker lacosamide, showed a weaker translational profile compared to duloxetine. In NCT00485472, oral administration (400 mg/day) failed to improve OA pain compared with placebo after 8 weeks of treatment, while preclinical MIA models demonstrated modest reductions in tactile allodynia and mechanical hyperalgesia, particularly at the higher dose at 7 and 14 days post OA induction^109^.

In summary, duloxetine demonstrated efficacy in both preclinical and clinical studies, whereas lacosamide showed efficacy only in preclinical models. All preclinical studies were conducted in MIA rat models, although different routes of administration and treatment initiation times were used. In contrast to clinical studies, where treatment was chronic and prolonged, preclinical efficacy was often assessed from minutes to hours after dosing. Only one preclinical study evaluated chronic administration over 28 days; however, treatment was initiated at the time of OA induction, which does not reflect the timing of patient enrollment in clinical studies. While chronic treatment with lacosamide failed to demonstrate efficacy in clinical trials, beneficial effects were observed in preclinical models. These discrepancies may be related to differences in treatment timing and frequency, as preclinical studies generally employed acute administration during the early phase of OA, as well as to the lack of assessment of spontaneous pain outcomes.

Details of the studies evaluating the efficacy of antidepressant and anticonvulsant drugs on OA pain are summarized in Table 4.

**Table 4:** Selected clinical and preclinical studies evaluating the efficacy of anticonvulsant and antidepressant, including study design, treatment characteristics, and pain-related outcomes

Drug	OA model/ affected joint	Dose	Administration route	Timing of treatment initiation	Outcome for pain assessment	Timepoint of efficacy assessment	Outcome: efficacy in pain reduction	References
Duloxetine	Knee	60–120 mg	p.o. / daily	7.44 ± 7.15 years	BPI	After 4, 7, 13 weeks of treatment	Efficacy superior to placebo	NCT00433290^103^
	Knee	60–120 mg	p.o. / daily	9.17 ± 8.47 years	11‑point Numerical Likert Scale Patient Diary	After 13 weeks of treatment	Efficacy superior to placebo	NCT00408421^103^
	Knee	60 mg	p.o. / daily	≥3 months	BPI, PGI‑I, CGI, HRQoL	After 14 weeks of treatment	Improvement of BPI vs placebo	NCT02248480^105^
	Knee	60 mg	p.o. / daily	≥3 months	BPI‑S, BPI‑I, PGI‑I, CGI‑I, SF‑36, WOMAC	Up to 48 and 52 weeks of treatment	Efficacy superior to placebo	NCT02335346^162^
	Knee/hip	60 mg	p.o. / daily	≥3 months	WOMAC	After 13 weeks of treatment	Efficacy superior to placebo	NCT01931475^106^
	Sprague Dawley rat / hip MIA	20 mg/kg	i.p. / single dose	28 days post OA induction	Mechanical allodynia	After 1 and 3 hours of treatment	Efficacy superior to the vehicle	^107^
	Sprague Dawley rats / MIA	30-150 umol/kg	p.o. / 20 minutes before the first test	20 days post OA induction	HGS	After 20 and 180 min of treatment	Efficacy superior to the vehicle	^39^
	Sprague Dawley rats / MIA	3 mg/kg	s.c./ single dose	14 days post OA induction	Mechanical hyperalgesia	After 3 and 24 hours of treatment	Efficacy superior to the vehicle	^74^
	Sprague Dawley rats / MIA	3, 10, 30 mg/kg	p.o. / single dose	21 days post OA induction	SWB, gait analysis	After 2 hours of treatment	Efficacy superior to the vehicle only for the SWB	^51^
	Wistar rat / MIA	15- 25 mg/ kg	p.o./ 28 days	0 day post OA induction	Thermal allodynia, SPB	Up to 28 days of treatment	Efficacy superior to the vehicle	^108^
Lacosamide	Knee	400 mg	p.o. / daily	≥6 months	WOMAC	After 8 weeks of treatment	Efficacy not superior to the placebo	NCT00485472 PNA
	Wistar rats / MIA	3, 10, 30 mg/kg	p.o. / 45–60 minutes before the test	3, 7, 14 days post OA induction	Mechanical allodynia, paw pressure test	After 3, 7, 14 days of OA induction	Improvement of tactile allodynia (highest dose) and mechanical hyperalgesia with all doses at 7 and 14 days	^109^

*BPI* Brief Pain Inventory, *BPI I* Brief Pain Inventory Interference, *BPI S* Brief Pain Inventory Severity, *CGI* Clinical Global Impression, *CGI I* Clinical Global Impression—Improvement, *HGS* hindlimb grip strength, *HRQoL* health-related quality of life, *i.a.* intra-articular, *i.p.* intraperitoneal, *MIA* monosodium iodoacetate, *PGI I* Patient Global Impression of Improvement, *p.o.* per oral, *SF-36* 36-Item Short Form Survey, *SPB* spontaneous behavior, *SWB* static weight bearing, *WOMAC* Western Ontario and McMaster Universities Osteoarthritis Index.

### Modulators of the transient receptor potential vanilloid 1

Capsaicin is classified as a counterirritant, a class of compounds that produces localized irritation or mild inflammation to relieve pain. Its analgesic activity is mediated through prolonged activation of the transient receptor potential vanilloid 1 (TRPV1) receptor, leading to desensitization of nociceptive sensory neurons^110^. Topical capsaicin formulations have been investigated clinically for OA pain management, particularly in knee OA. In the clinical trial NCT03528369, topical CGS-200 containing either 1% or 5% capsaicin was applied for 60 min daily over four consecutive days. The 5% formulation, but not the 1% formulation, met the primary efficacy endpoint by significantly reducing WOMAC pain scores at day 35 relative to baseline. Both formulations, however, demonstrated greater efficacy than vehicle treatment at later follow-up time points (days 64 and 94), suggesting prolonged analgesic effects. These findings have thus far been reported through ClinicalTrials.gov and congress abstracts.

A more targeted TRPV1 agonist formulation, CNTX-4975-05 (transcapsaicin), was evaluated in two clinical studies. In one trial (NCT03660943), repeated i.a. injections were assessed over 12 weeks using WOMAC outcomes, whereas a second study (NCT03429049) evaluated a single i.a. injection over 52 weeks using average weekly pain during walking as the primary endpoint. Neither study demonstrated superiority over placebo, indicating limited clinical efficacy for intra-articular transcapsaicin under these treatment conditions.

Preclinical findings in OA models revealed similarly heterogeneous outcomes. The studies employed MIA-induced OA in Sprague Dawley rat. In one study, intra-articular capsaicin administration (30–100 µg) rapidly improved mechanical hyperalgesia in the Randall–Sellitto test within 20 min after treatment, demonstrating acute antinociceptive efficacy against mechanically evoked pain^74^. Another investigation reported that prophylactic intra-articular administration of 0.5% capsaicin before MIA induction attenuated the development of OA-associated pain behaviors, including improvements in mechanical allodynia, weight-bearing asymmetry, and motor performance over four weeks^111^. In contrast, a separate study using intra-articular administration of 1 µg/µL capsaicin initiated after OA establishment failed to improve mechanical allodynia or weight-bearing deficits at multiple assessment time points^112^.

Details of the studies evaluating the efficacy of the modulators of vanilloid receptors on OA pain are summarized in Table 5.

**Table 5:** Selected clinical and preclinical studies evaluating the efficacy of modulators of vanilloid receptors, including study design, treatment characteristics, and pain-related outcomes

Drug	OA model/affected joint	Dose	Administration route	Timing of treatment initiation	Outcome for pain assessment	Timepoint of efficacy assessment	Outcome: efficacy in pain reduction	References
CNTX 4975-05	Knee	1 mg	i.a./twice	≥6 months	WOMAC	After 12 weeks of treatment	Efficacy not superior to the placebo	NCT03660943 PNA
	Knee	1 mg	i.a./single dose	≥6 months	Average weekly pain	After 52 weeks of treatment	Efficacy not superior to the placebo	NCT03429049 PNA
CGS‑200 capsaicin	Knee	1–5%	Topical/60 min per day, 4 days	≥3 months	WOMAC	After 35 days of treatment	5% (but not 1%) effective vs baseline	NCT03528369 PNA
	Sprague Dawley rats/MIA	1 ug/ul	i.a./single dose	7, 14, 21 and 28 days after OA induction	Mechanical allodynia, SWB	After 7, 14, 21 and 28 days from OA induction	Efficacy not superior to the vehicle	^112^
	Sprague Dawley rats/MIA	30–100 ug	i.a./single dose	14 days post OA induction	Mechanical hyperalgesia	After 20 min of treatment	Efficacy superior to the vehicle	^74^
	Sprague Dawley rat/MIA	0.5%	i.a./single dose	14 days before OA induction	Mechanical allodynia, SWB, SPB	Weekly for 4 weeks after OA induction	Efficacy superior to control group without treatment	^111^

Clinical trials are shown in grey rows, and corresponding preclinical studies for the same compounds are shown in white rows.

*i.a.* intra-articular, *PNA* publication not available, *SPB* spontaneous pain behavior, *SWB* static weight bearing, *WOMAC* Western Ontario and McMaster Universities Osteoarthritis Index.

In summary, both clinical and preclinical studies investigated knee OA and evaluated pain-related outcomes after local treatment administration. Yet, the efficacy results were inconsistent across models. Clinical studies generally failed to demonstrate superiority over placebo, except for the 5% topical formulation, which showed efficacy versus baseline only. In preclinical studies, one MIA study similarly reported no efficacy, whereas the other two studies demonstrated analgesic effects. However, in one of these positive studies, the lack of an appropriate vehicle-treated control group limits the interpretation of the findings, as efficacy was assessed only in comparison with the untreated MIA group rather than against a vehicle-treated control. The treatment regimens also differed substantially: patients received treatments with follow-up periods up to 12–52 weeks, while animal studies mainly employed single-dose administration with efficacy assessed either acutely or over shorter time frames. Another important discrepancy concerns the pain outcomes evaluated. Clinical studies primarily relied on functional and subjective measures such as WOMAC and average weekly pain, whereas preclinical studies mainly assessed evoked pain responses, including mechanical allodynia and hyperalgesia, with only limited evaluation of spontaneous pain-related behaviors.

### Biological therapies

Biological drugs are increasingly studied in OA to target inflammatory mediators that contribute to pain and joint degeneration^113^. Due to their species specificity, human-based biologics are needed to avoid immune reactions, which limit direct comparisons between human and animal studies.

NGF, a neurotrophin, signals via its receptors TrkA and the p75 common neurotrophin receptor (p75NTR) to induce chronic pain in OA. Fasinumab and Tanezumab are monoclonal antibodies targeting NGF. For fasinumab, subcutaneous administration (1–6 mg every 4–8 weeks) over 4–24 weeks significantly improved pain and functional outcomes compared with placebo in several studies (NCT03161093, NCT02683239, NCT03304379, NCT02447276, and NCT03285646)^114–117^. Intravenous single-dose administration also demonstrated efficacy at 8 weeks. Tanezumab similarly showed robust efficacy across multiple clinical trials (NCT00985621, NCT00863304, NCT00830063, NCT00744471, NCT00733902, NCT02528188, NCT02709486, NCT00669409, and NCT00399490), with improvements observed after intravenous or subcutaneous administration over treatment periods ranging from 8 to 72 weeks^118–126^.

Preclinical studies supported the analgesic efficacy of NGF blockade. Most investigations employed MIA-induced OA or medial meniscal transection models in rodents. Anti-NGF therapies consistently reduced mechanical hypersensitivity, improved weight-bearing asymmetry, and restored locomotor function in both rats and mice. In Sprague Dawley rat with MIA-induced OA, repeated subcutaneous administration significantly improved both spontaneous weight bearing and mechanical allodynia^127^. Similar improvements in gait and locomotor behaviors were observed in surgically induced OA models and after systemic or intra-articular administration. Additional studies further demonstrated improvements in burrowing activity, incapacitance test, and forelimb asymmetry, supporting effects on both evoked and spontaneous pain-related behaviors^43,71,73,128–132^. Other biological targets have shown weaker translational concordance. REGN5069, a monoclonal antibody targeting GDNF family receptor alpha-3, was developed to inhibit artemin-mediated pain sensitization. Although preclinical studies in DMM and MIA models demonstrated dose-dependent reductions in tactile allodynia and thermal hyperalgesia after prolonged subcutaneous administration^133^, the clinical trial NCT03956550 failed to demonstrate superiority over placebo in OA patients^133^. Similarly, LY2951742, a monoclonal antibody targeting calcitonin gene-related peptide, failed to improve WOMAC pain scores in OA patients during the clinical trial NCT02192190 despite encouraging preclinical efficacy^134^. In rat MIA and meniscal transection models, single subcutaneous administration produced prolonged reductions in pain-related behaviors and improvements in spontaneous weight bearing lasting up to 60 days^135^.

Details of the studies evaluating the efficacy of the biological drugs on OA pain are summarized in Table 6.

**Table 6:** Selected clinical and preclinical studies evaluating the efficacy of biological drugs, including study design, treatment characteristics, and pain-related outcomes

Drug	OA model/affected joint	Dose	Administration route	Timing of treatment initiation	Outcome for pain assessment	Timepoint of efficacy assessment	Outcome: efficacy in pain reduction	References
Fasinumab	Knee and hip	1 mg	s.c./every 4 and 8 weeks	≥3 months	WOMAC physical function subscale	After 16 weeks of treatment	Efficacy superior to placebo	NCT03161093^117^
	Knee and hip	n.a.	n.a.	n.a.	n.a.	After 16 weeks of treatment	n.a.	NCT03285646 PNA
	Knee	1, 3, 6 mg	s.c./every 4 or 8 weeks	≥3 months	WOMAC physical function scores	After 4, 8, 16 weeks of treatment	Efficacy superior to placebo without dose response	NCT02683239^116^
	Knee and hip	1 mg	s.c./every 4 weeks	≥3 months	WOMAC physical function scores	After 24 weeks of treatment	Efficacy superior to placebo	NCT03304379^114^
	Knee and hip	1, 3, 6, 9 mg	s.c./every 4 weeks for 12 weeks	n.a.	WOMAC	After 16 weeks of treatment	Efficacy superior to placebo without dose response	NCT02447276^115^
Tanezumab	Knee and hip	5–10 mg	i.v./single dose	n.a.	WOMAC pain	After 8 weeks of treatment	Efficacy superior to placebo	NCT00985621^118^
	Knee and hip	5–10 mg	i.v./single dose	n.a.	WOMAC pain and physical function	After 8 weeks of treatment	Efficacy superior to placebo	NCT00863304^119^
	Knee and hip	5–10 mg	i.v./single dose	n.a.	WOMAC pain and physical function	After 16 weeks of treatment	Efficacy superior to placebo	NCT00830063^119^
	Hip	2.5, 5, 10 mg	i.v./every 8 weeks	n.a.	WOMAC pain and physical function, PGA	After 32 weeks of treatment	Efficacy superior to placebo	NCT00744471^120^
	Knee	2.5, 5, 10 mg	i.v./every 8 weeks	Not available	WOMAC pain and physical function, PGA	After 16 weeks of treatment	Efficacy superior to placebo	NCT00733902^121^
	Knee	2.5, 5 mg	s.c./every 8 weeks	Not available	WOMAC pain and physical function, PGA	After 56 weeks of treatment	Efficacy superior to placebo	NCT02528188^122^
	Knee	10–200 ug/kg	i.v./single dose	Not available	WOMAC pain, physical function, and stiffness	After 8 weeks of treatment	Efficacy superior to placebo	NCT00669409^124^
	Knee and hip	2.5, 5 mg	s.c./every 8 weeks	Not available	WOMAC pain and physical function, PGA	After 24 weeks of treatment	Efficacy superior to placebo (5 mg)	NCT02709486^125^
	Knee	50 ug/kg	i.v./every 8 weeks	Not available	WOMAC pain and physical function, PGA	After 72 weeks of treatment	Efficacy compared to baseline	NCT00399490^126^
	C57BR/J mice/MIA	10 mg/kg	i.p./single dose	3 weeks post OA induction	Gait analysis	After 3, 4, 5 weeks from OA induction	Efficacy superior to vehicle	^128^
	Sprague Dawley rats/MIA	10 mg/kg	s.c./twice	2 and 3 weeks post OA induction	SWB, Mechanical allodynia test	Up to day 28 post-induction	Efficacy superior to vehicle	^127^
	Lewis rats/MMT	0.1, 1, 10 mg/kg	s.c./weekly for 3 weeks	0 day after OA induction	SWB	n.a.	Efficacy superior to the isotype control	^129^
	Sprague Dawley rats/MIA	1, 10, 100 µg/dose	i.a./single dose	14 days after OA induction	SWB, mechanical allodynia	After 6 weeks from OA induction	Efficacy superior to vehicle for weight bearing (only 100 µg dose) but not for allodynia	^130^
	C57BL/6 mice/MIA	10 mg/kg	i.p./single dose	3 weeks after OA induction	Gait analysis	After 3, 4, 5 weeks from OA induction	Efficacy superior to vehicle	^128^
	Sprague Dawley rats/MIA	0.1, 0.3, 1 mg/kg	i.v./single dose	3 days after OA induction	Gait analysis	After 35 days from OA induction	Efficacy superior to vehicle (0.3, 1 mg/kg)	^131^
	Wistar Han rats/bilateral MIA	9 mg/kg	s.c./single dose	3 days after OA induction	SPB	After 3, 14, 21 and 28 days of OA induction	Efficacy superior to vehicle	^71^
	Mouse/MNX	Endogenous anti‑NGF via vaccination	Vaccine inoculation/3 doses	Boosts at weeks 10, 12, 15 post surgery	SWB	Weekly up to 12 weeks after OA induction	Efficacy superior to the mock vaccine	^132^
	C57BL/6J/Mechanical loading	10 mg/kg	i.p.	1 and 7 days after OA induction	Gait analysis, mechanical allodynia	After 3 and 7 days of treatment	Efficacy superior to vehicle	^73^
	C57BL/6J/MIA	3 mg/kg	i.p./twice	2 and 4 weeks after OA induction	SWB, mechanical allodynia	Weekly up to 5 weeks after OA induction	Efficacy superior to the vehicle-treated group at week 4	^43^
REGN5069	Knee	100 or 1000 mg	i.v./3 times	Not specified	WOMAC pain subscale	After 12 weeks of treatment	Efficacy not superior to the placebo	NCT03956550^133^
	C57BL/6 mice/DMM or MIA	50 mg/kg	s.c./once or twice per week	0 day and 16 weeks (DMM) or 2 weeks (MIA) after OA induction	Mechanical allodynia	After 1, 3, 5 weeks after DMM or 2 weeks after MIA	Efficacy superior to the vehicle	^133^
LY2951742	Knee	5–300 mg	s.c./twice per week	Not specified	WOMAC	After 8 weeks of treatment	Efficacy not superior to placebo	NCT02192190^134^
	Lewis rats/MIA and MT OA	MIA: 1, 4, 16 mg/kg; MT: 0.25, 1, 4 mg/kg	s.c./single dose	14 days (MIA) or 16 days (MT) after OA induction	SWB	After 2 h (MIA) and up to 60 days (MT) from the treatment	Dose-dependent pain reduction in both models; duration up to 25 days (low dose) and 60 days (high dose, MT model)	^135^

*DMM* destabilization of the medial meniscus, *i.a.* intra-articular, *i.p.* intraperitoneal, *i.v.* intravenous, *MIA* monosodium iodoacetate, *MNX* medial meniscal transection, *MT* meniscal tear, *n.a.* not available, *PGA* Patient Global Assessment, *PNA* publication not available, *SPB* spontaneous pain behavior, *s.c.* subcutaneous, *SWB* static weight bearing, *WOMAC* Western Ontario and McMaster Universities Osteoarthritis Index.

The preclinical and clinical studies show a relatively good overall concordance, as most studies in both settings reported analgesic efficacy. However, important methodological differences remain. Clinical studies primarily involved patients with chronic symptomatic OA of the knee and/or hip, generally with symptoms persisting for at least 3 months, whereas preclinical studies often initiated treatment much earlier after OA induction, ranging from 0 days to 3 weeks. In some animal studies, treatment even began at the time of OA induction, which does not recapitulate the clinical condition of long-established disease. Another major discrepancy concerns treatment duration and follow-up. Clinical studies mainly evaluated chronic repeated administration over 8–72 weeks, while most preclinical studies used acute or short-term dosing schedules. Differences are also evident in the pain outcomes evaluated. Clinical trials relied predominantly on patient-reported outcomes such as WOMAC pain, stiffness, physical function, and PGA scores, which reflect multidimensional pain experience and disability. In contrast, preclinical studies mainly assessed evoked pain responses and locomotor alterations, including mechanical allodynia, gait analysis, and weight-bearing deficits, with fewer studies evaluating spontaneous pain behaviors such as SWB or SPB.

For both REGN5069 and LY2951742, a poor translational concordance emerges between preclinical and clinical findings. While preclinical studies consistently demonstrate analgesic efficacy in OA models, neither compound showed superiority over placebo in clinical trials. Differences are also present in treatment, timing, and duration. In preclinical studies, treatment was initiated relatively early after OA induction, ranging from the day of induction to 2–16 weeks afterward, depending on the model. Conversely, clinical trials involved established chronic OA, although symptom duration at the time of enrollment was not always specified. Additionally, the preclinical studies mainly assessed reflexive or spontaneous pain behaviors, whereas clinical trials evaluated multidimensional pain perception and physical function.

### Other small molecules

Additional therapeutic approaches for OA pain include senolytic agents, Wnt pathway modulators, bradykinin receptor antagonists, and lidocaine. UBX0101 is a senolytic small molecule designed to selectively eliminate senescent cells that contribute to OA progression and pain. Despite encouraging preclinical findings, clinical translation was unsuccessful. In Phase I and II trials (NCT04129944), single intra-articular injections of UBX0101 (0.5–4 mg/ml) in patients with symptomatic knee OA demonstrated acceptable safety and tolerability but failed to improve primary and secondary outcomes, including WOMAC pain, WOMAC function, and daily pain NRS scores, leading to discontinuation of development. Instead, preclinical studies in C57BL/6J mice subjected to ACLT-induced OA showed that repeated intra-articular administration beginning 14 days after surgery reduced cartilage degeneration and improved spontaneous weight bearing and thermal sensitivity^136^.

Lorecivivint (SM04690), a small-molecule modulator of the Wnt signaling pathway targeting CLK2 and DYRK1A kinases, has shown mixed clinical efficacy. Early clinical studies demonstrated improvements in pain and function, particularly in patients with unilateral or less severe OA. Phase IIa and IIb trials reported beneficial effects at the 0.07 mg dose, whereas subsequent Phase III studies failed to meet primary pain endpoints despite signals of efficacy in selected patient subgroups (NCT02536833, NCT02951026, NCT04385303, NCT03928184)^137–141^. Preclinical evidence was more consistent. In MIA-induced OA, a single intra-articular injection administered 3 days after OA induction significantly improved tactile pain thresholds and reduced weight-bearing asymmetry for up to 4 weeks^142^.

Bradykinin signaling through the B2 receptor (B2R) also contributes to OA-associated inflammation and pain sensitization. Fasitibant (MEN16132), a selective B2R antagonist, was evaluated in Phase II clinical trials (NCT02205814 and NCT01091116). Although pain improvement was observed across treatment groups, efficacy was not superior to placebo and no dose–response relationship emerged. In contrast, preclinical MIA-induced OA studies in rats demonstrated significant reductions in weight-bearing asymmetry lasting up to 10 days after a single intra-articular administration^143^.

Similar to Fasitibant, 12 weeks of topical lidocaine treatment did not demonstrate superior efficacy compared with placebo (NCT00589979). In contrast, intra-articular administration showed efficacy in alleviating spontaneous pain in both the MIA-induced rat model and the ACLT dog model^49,51,144^.

Details of the studies described in the previous paragraph are summarized in Table 7.

**Table 7:** Selected clinical and preclinical studies evaluating the efficacy of small molecules, including study design, treatment characteristics, and pain-related outcomes

Drug	OA model/affected joint	Dose	Administration route	Timing of treatment initiation	Outcome for pain assessment	Timepoint of efficacy assessment	Outcome: efficacy in pain reduction	References
UBX0101	Knee	0.5, 2, 4 mg/ml	i.a./single dose	n.a.	WOMAC pain and function, NRS	After 12 and 24 weeks of treatment	Efficacy not superior to the placebo	NCT04129944
	C57BL/6J/ACLT	1 mM	i.a./every 2 days for 2 weeks	14 days post OA induction	SWB, thermal allodynia	After 28 days of OA induction	Efficacy superior to the vehicle	^136^
Lorecivivint	Knee	0.03, 0.07, 0.23 mg	i.a./single dose	n.a.	WOMAC pain and function	After 13 weeks (26 and 52 weeks secondary timepoint)	Efficacy not superior to placebo; improvement at 0.07 mg in the subgroup with unilateral OA symptoms	NCT02536833^140^
	Knee	0.03, 0.07, 0.23 mg	i.a./single dose	n.a.	WOMAC pain, WOMAC function	6, 12, 24, 36, 48 and 60 months	Efficacy superior to the vehicle at 6 and 12 months in the 0.7 mg group	NCT02951026^141^
	Knee	0.03, 0.07, 0.15, 0.23 mg	i.a./single dose	n.a.	NRS, WOMAC pain, WOMAC function	12 and 24 weeks of treatment	Efficacy superior to the vehicle (0.07 and 0.23 mg)	NCT03122860 ^125^
	Knee	0.07 mg	i.a./single dose	>26 weeks	NRS, WOMAC pain	Up to 24 weeks	Efficacy superior to placebo in KL2 for NRS	NCT04385303^138^
	Knee	0.07 mg	i.a./single dose	>26 weeks	NRS	Up to 52 weeks of treatment	Efficacy not superior to the placebo	NCT03928184 ^137^
	Rat/MIA model	0.3 µg	i.a./single dose	3 days post OA induction	Mechanical allodynia, SWB	Up to 4 weeks of treatment	Efficacy superior to the vehicle	^142^
Fasitibant	Knee	3 doses (concentrations n.a.)	i.a./single dose	n.a.	WOMAC pain	After 2 weeks of treatment	Efficacy not superior to the placebo	NCT02205814 PNA
	Knee	0.125, 0.25, 0.50 mg	i.a./once or twice every 2 weeks	n.a.	WOMAC	Up to 3 months	Efficacy not superior to the placebo	NCT01091116 PNA
	Wistar rats/MIA	1, 3, 10 µg/25 µl	i.a./once or twice (after 2 weeks, only 3 µg/25 µl dose)	7 days after OA induction	SWB	After 1, 3, 7, 10, 14 days of treatment	Efficacy superior to the vehicle	^143^
Lidocaine	Knee	5%	Topical/daily	n.a.	PRS	Up to 12 weeks	Efficacy not superior to the placebo	NCT00589979 PNA
	Wistar rats/MIA	5 mg	i.a./twice	7 and 20 days after OA induction	Mechanical hyperalgesia, gait analysis and	After 3 and 20 days of treatment	Efficacy superior to the vehicle	^49^
	Sprague Dawley rats/MIA	1, 3, 10 mg	i.a./once	21 days after OA induction	Gait analysis	21 days after OA induction	Efficacy superior to the vehicle at 3 mg	^51^
	Dog/ACLT and unilateral focal impact injury	1%	i.a./once	3 months after OA induction	Gait analysis	After 3 months of OA induction	Efficacy versus contralateral knee	^144^

*ACLT* anterior cruciate ligament transection, *i.a.* intra-articular, *MIA* monosodium iodoacetate, *NRS* numerical rating scale, *PNA* publication not available, *SPB* spontaneous pain behavior, *PRS* pain rating scale, *SWB* static weight bearing, *WOMAC* Western Ontario and McMaster Universities Osteoarthritis Index.

Overall, these studies show limited concordance between preclinical and clinical findings, but methodological differences are evident. Clinical studies assessed efficacy in patients with established chronic OA using subjective and multidimensional outcome measures such as WOMAC pain, WOMAC function, and NRS scores, with follow-up periods extending up to 52 weeks. In contrast, preclinical studies evaluated pain-related behaviors such as spontaneous weight bearing, thermal allodynia, and mechanical allodynia, generally over shorter experimental periods ranging from days to weeks after OA induction. A major discrepancy concerns the timing of treatment initiation. In preclinical studies, treatment was administered relatively early after OA induction (3–14 days), corresponding to an early disease stage. Conversely, clinical studies involved patients with chronic OA. These differences, together with the route of administration as exemplified by lidocaine, may contribute substantially to the discrepancies in efficacy observed between preclinical animal models and clinical studies in patients.

This set of case studies highlights the recurring translational discrepancy between preclinical findings and clinical outcomes for most of the compounds considered. In the following section, we discuss the pitfalls in experimental animal models and propose refinements for study design to improve predictive validity.

## Refining experimental design for greater translational impact in OA pain

Pain is the primary symptom experienced by patients with OA and the main reason for seeking medical care^145^. The heterogeneity of OA endotypes, defined by distinct underlying mechanisms, coupled with the complex nature of pain, complicates the development of effective long-term treatments. The complexity is mirrored in the challenge of establishing translation-relevant animal models. In addition to the well-recognized difficulty of translating findings “from bench to bedside”, an equally important challenge lies in effectively translating insights “from bedside to bench”.

As outlined above, several discrepancies exist between preclinical and clinical study designs for all pharmacological classes. Although fundamental mechanisms of pain perception, such as nociception, are evolutionarily conserved between rodents and humans. Important differences arise in the central processing and modulation of pain signals. In particular, the organization of ascending pain pathways differs across species^146^, potentially limiting the predictive value of rodent models for therapies targeting central pain mechanisms. Species-specific differences are also evident for TRPV1 channel, which is expressed in approximately 32% of human sensory neurons, compared to about 78% of somatosensory neurons in rodents^147^.

Additional discrepancies concern the treatment timing, duration, and outcome assessment. Clinical studies primarily assess spontaneous pain and functional impairment, whereas preclinical studies often rely on evoked pain responses as the primary or sole endpoints evaluated. Among the paradigms used to assess spontaneous behavior in OA, gait analysis is the most widely employed, alongside measures such as burrowing and voluntary wheel running activity. An additional approach is to evaluate the rewarding effects associated with relief from ongoing pain. The conditioned place preference paradigm provides such an opportunity: animals are placed in a two-chamber apparatus and receive an analgesic in only one chamber; if the analgesic produces meaningful pain relief, the animal subsequently shows a preference for that chamber^30^. The use of non-evoked pain paradigms in chronic pain models has proven particularly important for assessing the efficacy of gabapentinoids and strong opioids, whereas these measures appear to be less critical for evaluating NSAID efficacy^148^.

The majority of animal research in OA pain relies on the MIA model, which primarily captures acute joint inflammation and cartilage damage. Although the MIA model does not fully recapitulate the temporal progression of joint damage observed in human OA^26^, this model has been shown to be a valid and widely used model for assessing the efficacy of pain-modulating interventions. Patients enrolled in clinical trials typically report pain that has persisted for at least 6 months, often for many years, reflecting a chronic condition characterized by gradual structural joint deterioration, altered bone architecture, changes in joint innervation, and the development of central sensitization. These features collectively give rise to a pain phenotype fundamentally different from the acute, inflammation-driven pain captured by the MIA model.

Consistent with this, the MIA model shows good concordance with clinical outcomes for therapies targeting inflammation, such as NSAIDs, opioids and corticosteroids, particularly in early stages of the disease. However, its predictive value declines in later disease stages, when chronic pain mechanisms dominate.

Another important consideration is population heterogeneity. In the preclinical studies described, most OA models were developed in Sprague-Dawley rats, an outbred strain characterized by greater genetic diversity. This choice may partially help address the excessive homogeneity often present in preclinical populations. In mouse studies, a similar approach could involve the use of outbred strains instead of the commonly employed inbred C57BL/6 mouse, potentially improving the translational relevance of the findings^149^. Indeed, outbred animals offer distinct advantages. Their genetic diversity more closely reflects human populations, in which variability in drug response is a key feature. In pain research, outbred mice exhibit a broader range of baseline nociceptive thresholds and more heterogeneous responses to injury, including those induced in OA models^150^. This variability should be considered biologically meaningful rather than experimental noise. It enables the modelling of clinically relevant subpopulations, such as responders versus non-responders, as well as mild versus severe pain phenotypes^150–152^.

Most of the people living with knee OA are women. Perimenopausal and postmenopausal women are overrepresented among affected individuals and report higher pain intensity compared to men. In addition, aging is an important factor in both sexes, as the majority of patients with OA, and consequently most participants in clinical studies, are over 60 years of age. In contrast, preclinical experiments typically use young, single-sex animal models. A substantial body of evidence demonstrates that the mechanisms underlying chronic pain differ between males and females, reinforcing the notion that a “one-size-fits-all” approach is insufficient for modeling OA pain or predicting therapeutic responses^153^. Pain research is frequently conducted in young rodents, largely due to the higher financial costs associated with housing or purchasing aged animals^149^. However, age influences not only the pharmacological mechanisms of certain treatments, such as senolytics, but also drug distribution, metabolism, gait, body weight, and fat composition^154–156^. Similarly, BMI and, even more importantly, the body fat composition, key characteristics of OA patients, are often overlooked. Most individuals with OA are overweight or obese, which contributes to a higher degree of systemic inflammation and increased mechanical stress on the lower joints. These factors, along with common comorbidities such as diabetes that may alter pain sensitivity, are typically not accounted for in preclinical studies^157^. Clinically, there is a bidirectional link between anxiety and pain that creates a vicious cycle: pain triggers anxiety, which in turn amplifies pain perception, complicating treatment outcomes. Therefore, assessing and managing anxiety is crucial in both preclinical and clinical pain studies, as failure to account for affective states may confound treatment efficacy evaluations.

Translational relevance is further influenced by the route and frequency of drug administration. These should reflect the compound’s mechanism of action, tissue distribution and clinical feasibility. For example, TRPV1-targeting compounds act on nociceptive fibers not located in cartilage itself, but in innervated joint tissues such as synovium, capsule and periarticular structures. Therefore, topical or periarticular delivery may be appropriate for compounds such as capsaicin, whereas intra-articular delivery may be required for compounds intended to access synovial or capsular nociceptor terminals. However, intra-articular strategies are limited by rapid clearance, restricted tissue diffusion and short residence time, making dosing schedules particularly important. Notably, some preclinical studies employ dosing regimens that are not clinically feasible, such as high-frequency intra-articular injection. For example, the senolytic UBX0101 showed efficacy in mouse OA models under repeated dosing schedules, including six injections every 2 days, which differed substantially from those evaluated in clinical trials. Thus, preclinical efficacy should be interpreted in relation not only to compound activity, but also to whether the administration route and dosing frequency are clinically plausible^158^.

Substantial research is being conducted in parallel within the veterinary field of pain management in companion animals with OA. Among these, dogs, cats, and horses are the most affected species, offering a unique opportunity to investigate OA pain in models of spontaneously occurring disease. These naturally occurring models more closely resemble the human condition, as animals are exposed to heterogeneous environments, levels of physical activity, and dietary factors comparable to those experienced by humans. Moreover, validated methods for assessing spontaneous pain-related behaviors are available in these species^159^. As highlighted by Duncan Lascelles and colleagues, companion animal models can complement traditional laboratory animal studies. For example, tissues obtained from these animals may be used to reduce the translational risks. In addition, evaluating drug efficacy in companion animals, potentially parallel with early clinical safety studies in humans, may provide valuable information to support decision-making on progression to Phase II clinical trials to reduce the failure rates of human clinical trials^160^.

Finally, we identified multiple drugs tested in humans for which data on animal studies were not available. While this may reflect unpublished studies, it highlights a lack of transparency that limits cumulative scientific progress. Ensuring accessibility of preclinical data is essential for improving reproducibility and informing translational strategies.

## Conclusions

**Fig. 2:**
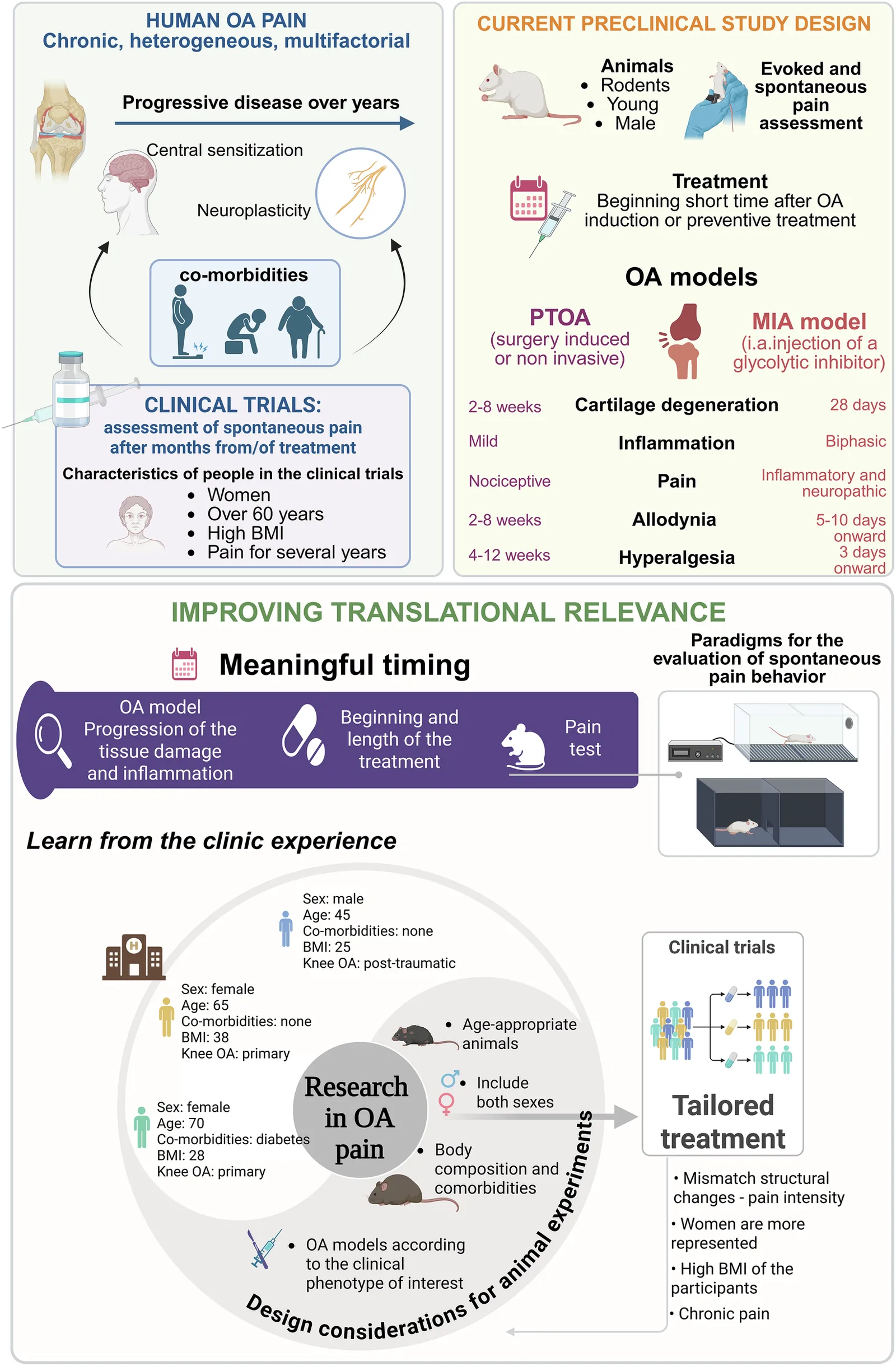
Improving translational relevance in OA pain research. Comparison of key characteristics between clinical trial populations (top left panel) and preclinical OA models (top right panel). The figure emphasizes the importance of clinically relevant timepoints for therapeutic administration and pain behaviour assessment in laboratory animal models, and the implementation of paradigms for the evaluation of spontaneous pain behaviour. Moreover, identifying patient phenotypes and recreating them in preclinical models may enhance translational relevance and support the development of tailored treatments (bottom panel). OA osteoarthritis, BMI body mass index, PTOA post-traumatic osteoarthritis, MIA monoiodoacetate, DMM destabilization of the medial meniscus, MNX meniscectomy, ACLT anterior cruciate ligament transection, i.a. intra-articular.

This review aimed to realign preclinical OA pain research with clinical reality. By comparing clinical trials with corresponding animal studies, we identify both areas of concordance and key translational gaps. Given the complexity of OA, it is not feasible to model all patient characteristics within a single preclinical system. Instead, experimental design should be guided by clearly defined target populations and clinically relevant phenotypes. While establishing a clear correspondence between pre-clinical model characteristics and human endotypes is not possible due to limited information on endotype-specific features, achieving consensus on correlations with clinical phenotypes is both feasible and necessary. The timing of drug administration and the time points selected for pain assessment should be appropriate and translationally relevant. Specifically, as in human OA, the preclinical model should recapitulate the structural and inflammatory changes that progressively lead to pain and allodynia. Drug treatment should therefore be initiated after the establishment of these pathological features, thereby mimicking a therapeutic intervention rather than a prophylactic approach. Moreover, pain assessment should include spontaneous pain-related behaviors, such as gait analysis and static weight bearing, to more closely mimic the functional and pain-related outcome measures commonly used in clinical practice (Fig. 2).

Refining experimental approaches in this way is not only an ethical requirement aligned with the Replacement, Reduction, and Refinement principles but a scientific necessity. Tailoring models to reflect the clinical reality will substantially enhance the translational value of preclinical research and ultimately improve the predictive power for clinical outcomes.

## Supplementary information

Transparent Peer Review file

Supplementary information
