# Supplementary material for: Assessing the translational value of animal models in developing pain-targeting therapies for clinical osteoarthritis
Source: Commun Med (Lond). 2026 Oct 8;6:522. doi: 10.1038/s43856-026-01926-7 (PMC13649651; doi:10.1038/s43856-026-01926-7)
Supplement: Supplementary file 1 [file 43856_2026_1926_MOESM1_ESM.pdf]

nature portfolio

Peer Review File

Assessing the translational value of animal models in developing pain-targeting therapies for clinical osteoarthritis

Corresponding Author: Dr Carmen Corciulo

This file contains all reviewer reports in order by version, followed by all author rebuttals in order by version.

Version 0:

Reviewer comments:

Reviewer #1

(Remarks to the Author)
Stubelius and co-authors present a narrative review discussing the translational value of in vivo studies for development of treatments targeting pain in osteoarthritis. The topic is important and timely, and there are many interesting facts listed in this review. However, there are major concerns:

General Comments
1. The selection of the references included is not clearly articulated, both for the clinical trials as well as for the animal studies. For trials, it is stated that clinicaltrials.gov was searched, but the references included seem somewhat random. For example, for an important topic such as i.a. corticosteroids, two rather random references are provided (ref. 33 and 34). Better search criteria would be helpful. For the animal studies, the selection of models is not clear. Fig 1 specifies n=104 clinical studies with corresponding animal studies, but then many non-OA models are discussed, and it is not clear if those are all part of the n=104. The n=104 should be better described and inclusion criteria defined. For example, lines 221–230 discuss testing of SI-613 in a model of CIA in rats, while CIA is not an OA model but an RA model. A model using silver nitrate–induced OA is discussed, which is an atypical (and uncommon) OA model; it is more a subacute arthritis that is inflammatory in nature. For ref. 22, no pain results are discussed, only inflammation and cartilage (in a non-OA model).
2. Further, there appear to be errors in the discussion/interpretation of certain references, e.g., ref. 27 is referred to as a carrageenan-induced paw edema (line 245) whereas in reality it is a murine DMM model; ref. 28 is discussed as a neuropathic model and line 247 says “OA models were not available” while ref. 28 is a genuine OA model (ACLT) with solid pain outcomes.
3. Translational relevance of certain pain outcomes is not always clear. For example, line 238, ref. 25, discusses heat sensitivity as an outcome in the MIA model, but is that translationally relevant? Have alterations in heat sensitivity been described in patients living with OA?
4. Frequently, specificity is lacking. As the subject of the review is the importance of OA model choice, it is not appropriate to omit details around the model, such as Line 251, “In a rabbit OA model, celecoxib…”. Likewise, the details of pain evaluation in all mentioned clinical and preclinical must be described thoroughly. For instance, lines 409-411: “where the preclinical data suggested a synergistic mechanism of action.” Also lines 447-449. Please check the review for specificity.
5. There are many unfinished and unsupported statements. For instance, statements regarding companion animals are vague and unreferenced. These points need to made clear with concrete evidence and it is suggested to only discuss in section 3. As another example, specific points related to the benefit of using outbred mice should be reviewed from reference 101 and should be presented (Line 526).
6. The illustrative figures (2 and 3) are unclear and lacking relevancy. As the theme of the review is whether preclinical trials relate to clinical successes/failures, the figures/tables should clearly synthesize and present this knowledge. For instance, this may be achieved with a main table that clearly lists the details of the preclinical model used (e.g. DMM, preclinical outcome, clinical trial details, clinical trial outcomes (WOMAC improvement/no sig. dif.).
7. The authors should synthesize and report their findings regarding best preclinical pain evaluation method. For instance, might it be said that tactile allodynia led to less instances of predicting clinical trial success, as 3/3 studies discussed did not show significance in clinical trials compared to placebo? Points such as these should be discussed in Section 3. A synthesized figure or table as suggested above would assist in the saliency of these claims.

Specific Comments

1. Line 101, please specify ref 8 is about knee OA.
2. Line 152: the von Frey test assesses mechanical allodynia, not hyperalgesia.
3. Line 170: please provide a ref for this statement.
4. The DOI hyperlink for ref 23 leads nowhere.
5. Line 258: this was also true for diclofenac. The result is not interpreted. What does it mean if it only affected allodynia 3 days after induction? How does this relate to the clinical trials?
6. Line 294: here again, there's insufficient information provided around the model, and the results are not interpreted with respect to translational value.
7. Ref 42 is not correct
8. Statement in line 312 is missing a reference
9. Line 321: define analgesic activity.
10. Ref 60: this was hip OA- please discuss and provide details on the analgesic activity and what it means.
11. Line 342: "in contrast" is not appropriate here. Mechanical hyperalgesia cannot be contrasted to the WOMAC. Also, ref. 61 also measured allodynia.
12. Line 357: please provide refs. for the CNTX-4975-05 discussion
13. Grammar mistakes, including incomplete sentences should be fixed. Line 80: "Especially pain remains a major translational hurdle". Line 507, spelling in Figure 3.
14. Line 223-224: The treatment showed chondroprotective effects relative to what? Vehicle or Diclofenac?
15. Figure captions need more descriptions and abbreviations.
16. Spelling line 7: "on behalf"
17. Line 152: von Frey – no capital in "von".

Reviewer #2

(Remarks to the Author)
This review comprehensively summarizes, from the standpoint of the working group to which the authors belong, the limitations of current animal models in the development of novel analgesics targeting osteoarthritis (OA) pain. In particular, the collection of clinical trial data from ClinicalTrials.gov and the alignment of these data with findings from animal studies are valuable contributions.
However, the current format of data presentation and the comparison framework combine efficacy and safety in a way that makes it difficult for readers to grasp the overall picture.

The authors selected clinical trials and provided them as a supplementary table, but the results should be presented in a format that facilitates reader understanding—for example, a summary table compiling the investigated items (drug, route of administration, inclusion criteria, evaluation period, primary outcomes, etc.).

Although the manuscript states that the 104 selected trials are those for which corresponding animal model data exist, these results are not shown. A summary table of the surveyed animal model data should also be created and presented.

In Section 2.2 ("Case studies"), the authors contrast results from clinical trials with those from animal models and describe their differences in a descriptive manner. To enhance reader comprehension, the corresponding clinical and nonclinical results should be organized into a comparative table. This table should specify which animal models were used (e.g., MIA model, rat strain, age, body weight, sex) and the methods of pain assessment (e.g., weight bearing, von Frey), together with the corresponding clinical trial outcomes.

The discussion focuses primarily on the limitations of OA pain animal models. However, anti-NGF antibodies have demonstrated efficacy in both animal models and clinical settings, indicating that the current animal models can evaluate efficacy at least to some extent. Conversely, TRPV1 antagonists show efficacy for postoperative pain in animal models, whereas their clinical efficacy for postoperative dental pain is reported to be limited. This suggests that the issue is not only limitations in OA models but also potential species differences in the function of the drug target itself in pain mechanisms. The authors should discuss species differences in target biology related to pain, supported by relevant literature.

The discrepancy between expected effects from animal models and insufficient efficacy in clinical pain trials is not limited to OA pain; it is also observed in the development of drugs for neuropathic pain and other pain conditions. This stems fundamentally from the subjectivity of "pain" in clinical settings, which cannot be directly assessed in animals. While the authors conclude that future work should focus on improving OA pain animal models, they should also discuss the broader issue of translational challenges in pain itself, along with possible solutions (e.g., brain imaging, experimental human model).

Reviewer #3

(Remarks to the Author)

Summary of the manuscript

This review article aims to examine the translational gap underlying the failure to develop effective pain-targeting therapies for clinical osteoarthritis (OA). The authors introduce the scale of the problem, highlighting some of the difficulties in recapitulating complex multifaceted diseases in simplified animal models, and then state the aim of critically retracing the path from clinical observations back to animal models, and proposing strategies to enhance translational relevance. Mechanisms of OA in humans and animals, and differences in methods of assessing of pain are discussed.

The overall approach of the review is to identify completed phase 2 & 3 clinical trials with OA pain as the primary end point, and to compare the findings to corresponding published preclinical studies using the same agents. The aim is to identify potential reasons underlying clinical failure of some analgesics. Clinical studies are categorised based on the pharmacological intervention (NSAIDs, corticosteroids, opioids, antidepressants and anticonvulsants, TRPV1 modulators, biologic drugs, and other small molecule inhibitors), and compared to the preclinical literature. The authors then make suggestions for refining experimental design to improve translatability, concluding that future research should focus on tailoring animal models to specific patient populations, rather than using general disease models.

Overall impression of the work

This is an important area for review, particularly given the recent announcements from the UK government and FDA of policies aiming to phase out and replace animals in scientific research. A review of how well animal models align with clinical studies in OA is therefore timely. It is however surprising that the role of novel non-animal methodologies to address the translational gap is not considered (see comment 3 below).

The approach of tracing clinical trials back to the preclinical literature to identify the reasons for differences in effectiveness of treatments is interesting, and the authors comprehensively collate completed trials. However, they provide less detailed coverage of preclinical studies and the level of citations is weak in places, in addition the two are not always well linked. The overall aim of this review is unclear as it is variously stated as “critically examining how current in vivo models align – or fail to align – with the clinical reality of OA pain in humans” (page 4 line 86) and “to assess the design and outcomes of clinical trials, with a specific emphasis on discrepancies relative to preclinical animal data” (page 8, line 173)- it si not clear what they mean by ‘discrepancies relative to preclinical animal data’. A consistent overall aim would help to focus the manuscript, which currently does not meet either of these statements, and there are several other areas for improvement.

Specific comments and recommendations

1. Although the focus is on analgesic development for OA pain, the review would benefit from a wider perspective of other chronic pain states, such as neuropathic pain states and relevant experimental models. This will ensure that the audience is aware that there are similar translational gaps for other chronic pain states. They could then highlight some key common areas relevant to the underlying basis for the translational gap. Areas that could be considered for inclusion: heterogeneity of clinical pain conditions, the complexity and diversity of underlying pathophysiological mechanisms, and the unreliability of some preclinical pain models. Lack of predictive validity (discussed in Yekkirala et al. 2017); discordance between preclinical and clinical testing - heterogeneity in clinical populations vs homogeneous animal subjects, types of pain mechanisms studied (discussed in Yang et al., 2025); poor alignment of clinical and preclinical endpoints, need more translational endpoints, greater importance of spontaneous (non-evoked) pain behaviour (discussed in Fischer et al 2021, Huerta et al., 2025).

2. Description & discussion of animal models – The authors state that a critical issue with animal models is that they do not mimic the full complexity of OA in humans (line 79). Whilst this is undoubtedly true, it is also well-recognised that OA is a heterogeneous clinical diagnosis which likely represents a collection of diseases, which can be stratified into different phenotypic presentations with varying underlying pathologies. The majority of induced models of arthritis aim to mimic key aspects of the disease (e.g. OA-like pain) or specific interactions (e.g. co-morbid anxiety an OA-like pain), rather than the disease state as a whole, which is in line with the authors own recommendations (Figure 3). There are numerous examples of back-translation of clinical phenotypes in the literature, and these should be cited.

The authors mention the potential utility of OA in companion animals (line 126, line 589), but do not elaborate or provide supporting references. These should be included, and the suggestion of greater involvement of, and consultation with, veterinarians expanded upon. It would also be worth including some of the extensive literature on spontaneous OA in Dunkin-Hartley guinea pigs (Jimenez, Glasson et al. 1997) as a more ethologically relevant model, and the potential for opportunistic preclinical studies of OA in larger animals such as pigs, sheep, goats, & horses (Stewart, Gilbert et al. 2024) as a means to circumvent issues of cost in ageing research animals up for clinical relevance to the chronic pain seen clinically (lines 532-537).

The statement that the rodent MIA model of OA as not reproducing the structural joint changes of OA (line 146) is not supported by the literature. Numerous studies have identified clinically relevant structural changes in the MIA model, including synovium, cartilage, and subchondral bone, and this should be acknowledged. Similarly, the authors state that to model the heterogeneity of the human pain experience, outbred strains of rodents should be used, rather than inbred C57BL/6 mice. This ignores the fact that many studies are conducted using outbred rat strains such as Sprague Dawley and Wistar, which are notably genetically heterogeneous (Gileta, Fitzpatrick et al. 2022)

In later descriptions of studies, key details of the models, strain and sex of animals, and endpoints measured are lacking. Given the aim of the review to investigate the relevance of preclinical models to clinical populations and to identify reasons for the translational gap, it is important to describe how each model works, and what aspect of OA it is modelling. For example, when the effects of diclofenac are described in three models of arthritis (page 10, lines 221-230), it is not clear how arthritis is induced in the silver nitrate model in male Sprague Dawley rats, and no details are provided on what constitutes the rabbit model of arthritis (line 229) or which sexes were involved. This is also true of the rabbit OA model in which

celecoxib was tested (line 251) and strain of rats used for testing melocoxicam (Wistar, line 236). Often multiple models are discussed without clearly delineating the differences (e.g. ACLT, pMMx & MIA in rats, line 469). Section 1.3 could be expanded to include brief descriptions of these models, or they could be introduced individually when first described.
3. Description of differences in pain assessment (Page 7, line 149-157). The review of the assessment of pain behaviour would benefit from greater detail and appropriate referencing. The excellent review by Zaki (2025) provides considerable detail on selection of model, consideration of outcome measures and relevant factors to consider when designing a preclinical study. Greater referencing of this review would be beneficial. This section could be developed to highlight opportunities to provide more translatable measures of pain in OA (Tappe-Theodor, King et al. 2019). For example, quantitative sensory testing (QST, line 169) is analogous to assessment of withdrawal thresholds in preclinical models & provides an opportunity to more closely translate findings between the two. This section could be developed by linking to studies that have used temporal summation of QST responses to identify neuropathic pain components in OA patients, with predictive power for determining incidence of pain persistence following total joint replacement. Whilst preclinical studies cannot replicate the subjective measures of pain intensity reported in the clinic, use of paradigms such as conditioned place preference, and studies that have attempted to back-translate conditioned pain modulation (CPM) testing in pain patients to preclinical studies should be mentioned.

4. The overall level of detail (and supporting references) will aid comprehension and improve the quality of the review. For example- page 5, line 120-123- clearer description of the differences and provide the supporting evidence. Page 5/ 6 (line 125-129) the utility of companion animals- inclusion of relevant citation required. In addition, any limitations (study design, controls, objective endpoints) in the study design should be included to ensure balance in the narrative (see above point).

Page 6, (line 144-147): The discussion of the MIA model requires citations supporting the statement that it doesn't reproduce structural changes seen in OA (see point 2). There are multiple papers from multiple groups reporting changes to cartilage, bone and synovium and therefore it would be useful for the authors to be clear which structural joint changes are not reproduced whilst providing balance in the reporting of those features that are exhibited and clinically relevant. The review cited Zaki et al. 2025 does state that the MIA model may not recapitulate the pathophysiology of OA or the molecular changes seen in humans. The second review cited here (Rosi et al 2022, which is authored by one of the co-authors herein) doesn't appear to mention the MIA model in any detail and therefore isn't ideal for this citation. Missing references – some statements are unsupported by references, e.g. studies in the DMM model which have predictive power for the joint safety of opioids & anti-NGF treatments, lines 541-5.a

5. Alternative methodologies to animal models - Given the recent announcements from the UK government (https://www.gov.uk/government/publications/replacing-animals-in-science-strategy) and FDA (https://www.fda.gov/news-events/press-announcements/fda-announces-plan-phase-out-animal-testing-requirement-monoclonal-antibodies-and-other-drugs) of policies aiming to replace animals in scientific research, it is important for reviews in this area to consider the contribution of new approach methodologies, such as 3D multi cell-type culture systems, & organs on chips, particularly those produced from relevant patient iPSCs, or computation models in replacing some aspects of animal models. E.g. recent commentary in nature (Kwon 2026). Whilst this is not the major focus of the review, discussion of how this applies to complex multi-system/level diseases such as OA and recent studies that have attempted this (Salgado, Jordan et al. 2021, Ummarino, Pensado-López et al. 2023, Marrero - Berrios, Salter et al. 2024) would be a welcome addition.

6. Effect sizes – In order to compare efficacy of drugs between clinical & preclinical studies, it is necessary to give an indication of the effect sizes reported, rather than just stating that, for example, a treatment reduces pain. This can be challenging when measurements of pain are not equivalent (e.g. self-reported pain in humans and withdrawal thresholds in rodents), but an indication of relative efficacy would still be useful. This will also aid in comparing between different classes of compounds.

7. The contribution of neuropathic pain mechanisms (Page 5, lines104-107). This requires some more consideration. The sentence line 104-106 relating to stretched or compressed articular nerves requires a reference and the authors need to be clear on what is meant by the statement 'emergence of symptoms recognised as neuropathic pain'. The reference cited (Hoffman et al. 2025) does not discuss neuropathic pain, it does refer to neuroplasticity, but this can be a mechanism contributing to nociceptive and neuropathic pain. The IASP definition of neuropathic pain provides a clear framework to support distinctions between possible underlying mechanisms.

8. Relationship between pain and anxiety and impact on treatment outcomes. Page 5 (lines 116-119) discusses comorbidities and influences on pain perception, this could be expanded to discuss anxiety and the impact of anxiety on chronic pain and treatment responses. This is relevant to the phenotyping of OA pain, mapping to pain mechanisms which informs treatment selection (Figure 3 -tailored treatment).

9. Consistency between findings (clinical versus pre-clinical). The authors correctly identify that there is consistency for NSAIDs, this is also the case for opioids-but this isn't highlighted in the review and could be expanded upon. There is also consistency for anti-NGF treatments. Page 17 (lines 386-391) mentions one study, there are multiple other examples where anti-NGF treatments were shown to have beneficial effects in rodent models of OA pain (PMID: 29100502, PMID: 27208420, PMID: 25677108, PMID: 25966657, PMID: 30862648) - the rationale for the study selected isn't clear, and greater balance would be provided by a broader group of citations.

10. Conclusions – the concluding statement reads more as a list of limitations and doesn't summarise the overall findings and recommendations of the review, which are not entirely clear. From Figure 3, it appears that a major suggestion is to back-translate clinical phenotypes to more specifically model patient populations, particularly the use of female rodents with

comorbid obesity and/or diabetes to achieve this. This proposal would be strengthened by the inclusion of studies which have already undertaken this type of more complex modelling. The use of a combination of models including those that mimic different facets of clinical OA in multiple species would appear to be a fitting conclusion.

The conclusion discusses both human endotypes and clinical phenotypes and importantly in the context of this review the 'limited information on endotype specific features'. It would be beneficial if the authors were clearer on the meaning of terms endotype and phenotype in the context of OA pain mechanisms, as they appear to be inter-changeable. Our reading of this section is that lack of 'consensus in the clinical phenotypes' still needs to be overcome so that it is clear which clinical phenotypes need to be modelled to address the translational gap in the development of new analgesics for OA pain. This may also be a fitting conclusion.

Formatting issues & minor suggestions
Article title – the current phrasing is a little unclear, and does not fully represent the aim of the review, suggest an alternative along the lines of:
Assessing the translational value of animal models in developing pain-targeting therapies for clinical osteoarthritis
Line 154 – the open field test is not considered to be ideal for assessing spontaneous locomotor activity as it can be considered anxiogenic, and this may interfere with interpretation in conditions such as OA where anxiety and physical disability are comorbid. Locomotor activity measurement systems, or those that combine observation of naturalistic behaviours and gait parameters would be preferable.
Figures - figure 2 is titled as a graph, but would be better described as an infographic. It has potential to be useful to the reader, but a comprehensive legend should be provided containing all the information required to understand it. This includes definitions of acronyms and abbreviations, and a narrative description of what the authors are trying to explain. It is not currently clear how the structural phenotypes listed at the top relate to the pain mechanisms in the bottom 2 panels, and the sites of actions of pharmacological treatments are incomplete or unclear. Figure 3 could be improved by refinement of the text on the graphic – it is not clear what the obesity OA model is, or how mismatch structural changes - pain intensity, women are more represented, high BMI of patients, and chronic pain feeds back into the preclinical models (grey arrow, lower right-hand side) – and a comprehensive figure legend.
Acronyms & abbreviations should be defined at first usage, e.g. CGRP in line 391 rather than 443.
Formatting – italics for latin (e.g. in vivo, line 547, 599)
References – numerical citations in the text should appear before full stops rather than after.
One of the supplementary tables (8037_0_supp_184648_t7qckt) does not have a title, explanation, or table number
References
Fischer AS, Lanigan MT, Upton N, Lione LA (2021) Preclinical neuropathic pain assessment: the importance of translatability and bidirectional research. Frontiers in Pharmacology 11, 614990
Gileta, A. F., C. J. Fitzpatrick, A. S. Chitre, C. L. St. Pierre, E. V. Joyce, R. J. Maguire, A. M. McLeod, N. M. Gonzales, A. E. Williams, J. D. Morrow, T. E. Robinson, S. B. Flagel and A. A. Palmer (2022). "Genetic characterization of outbred Sprague Dawley rats and utility for genome-wide association studies." PLOS Genetics 18(5): e1010234.
Huerta MA, Roza P, Cisneros E, Alique M, Roza C (2025). Enhancing clinical translation of analgesics for neuropathic pain: a systematic review and metaanlysis on the role of non-evoked pain assessment in preclinical trials. Pain 166(11) 2473-2486.
Jimenez, P. A., S. S. Glasson, O. V. Trubetskoy and H. B. Haimes (1997). "Spontaneous osteoarthritis in Dunkin Hartley guinea pigs: histologic, radiologic, and biochemical changes." Lab Anim Sci 47(6): 598-601.
Kwon, D. (2026). "The age of animal experiments is waning. Where will science go next?" Nature 650(8103): 812-814.
Marrero - Berrios, I., S. E. Salter, R. Hirday, C. P. Rabolli, A. Tan, C. T. Hung, R. S. Schloss and M. L. Yarmush (2024). "In vitro inflammatory multi-cellular model of osteoarthritis." Osteoarthritis and Cartilage Open 6(1): 100432.
Salgado, C., O. Jordan and E. Allémann (2021) "Osteoarthritis In Vitro Models: Applications and Implications in Development of Intra-Articular Drug Delivery Systems." Pharmaceutics 13, 60 DOI: 10.3390/pharmaceutics13010060.
Stewart, H. L., D. Gilbert, D. Stefanovski, Z. Garman, M. B. Albro, M. Bais, M. W. Grinstaff, B. D. Snyder and T. P. Schaer (2024). "A missed opportunity: A scoping review of the effect of sex and age on osteoarthritis using large animal models." Osteoarthritis and Cartilage 32(5): 501-513.
Tappe-Theodor, A., T. King and M. M. Morgan (2019). "Pros and Cons of Clinically Relevant Methods to Assess Pain in Rodents." Neuroscience & Biobehavioral Reviews 100: 335-343.
Ummarino, A., A. Pensado-López, R. Migliore, L. Alcaide-Ruggiero, N. Calà, M. Caputo, F. M. Gambaro, C. Anfray, F. L. Ronzoni, E. Kon, P. Allavena and F. Torres Andón (2023). "An in vitro model for osteoarthritis using long-cultured inflammatory human macrophages repeatedly stimulated with TLR agonists." European Journal of Immunology 53(12): 2350507.
Yang J, Xie, Y-F, Smith R, Ratte, S, Prescott, SA (2025) Discordance between preclinical and clinical testing of NaV1.7-selective inhibitors for pain. Pain 166 (3) 481-501.
Yekkirala AS, Roberson DP, Bean BP, Woolf CJ (2017) Breaking Barriers to novel analgesic drug development. Nature Rev Drug Discov 16 (8) 545.

Vicky Chapman and Stephen Woodhams, Arthritis UK Pain Centre, University of Nottingham

Version 1:

Reviewer comments:

Reviewer #2

(Remarks to the Author)
The authors have appropriately addressed many of the reviewers' comments. However, several statements described below do not appear to be fully supported by, or consistent with, the findings of the present review. The authors are requested to carefully reconsider these points.

Comment 1 (Discussion, Lines 604–605)
In the Discussion (Lines 604–605), the authors state that “While the MIA model is valuable for studying acute inflammatory processes, it does not mirror the slow and progressive trajectory of human OA.” However, many of the findings presented in this review demonstrate substantial concordance between the clinical outcomes and the results obtained in the MIA model. Therefore, the data summarized in this manuscript do not appear to support this statement.
Furthermore, Reference 26 also indicates that the MIA model reflects key aspects of clinical OA, which is inconsistent with the above assertion. The authors should provide clear evidence and justification supporting this statement or revise the discussion accordingly.

Comment 2 (Discussion, Lines 611–614)
In the Discussion (Lines 611–614), the authors state:
“Consistent with this, the MIA model shows good concordance with clinical outcomes for therapies targeting inflammation, such as NSAIDs, opioids and corticosteroids, particularly in early stages of the disease. However, its predictive value declines in later disease stages, when chronic pain mechanisms dominate.”
However, as described by the authors themselves in the opioid section, opioids are generally used for “moderate-to-severe symptoms insufficiently controlled by first-line therapies,” indicating that opioids primarily target chronic pain in more advanced stages of OA rather than inflammation.
Therefore, including opioids as examples of anti-inflammatory therapies is inappropriate, and the statement regarding the predictive limitations of the MIA model in later disease stages is not supported by the findings presented in this review. In fact, the current analysis shows that opioids demonstrate efficacy in the MIA model that is largely consistent with their clinical efficacy. Accordingly, this section should be revised.

Comment 3 (Figure 2: MIA Model)
In Figure 2, cartilage degeneration in the MIA model is indicated as occurring at 28 days. However, the basis for this specific time point is not provided anywhere in the manuscript.
Since the purpose of Figure 2 is to summarize the findings of the present review, all information presented in the figure should be directly supported by the data collected in this study. The authors should therefore add a new column to Tables 1–7 indicating whether cartilage degeneration was observed in each animal model reviewed, and summarize the findings derived from those data.
In addition, the timing of allodynia and hyperalgesia should be revised. Based on the literature reviewed, it would be more appropriate to indicate allodynia as occurring 'from days 5–10 onward' and hyperalgesia 'from day 3 onward'.

Comment 4 (Figure 2: Learn from the Clinical Experience)
In the “Learn from the Clinical Experience” section of Figure 2, the authors suggest that a 3-month-old ACLT model is appropriate for modeling OA in males and that a 7-month-old animal model is appropriate for females. However, the manuscript does not provide any supporting evidence for these recommendations.
As Figure 2 should reflect the findings of the present review, the authors should add additional columns to Tables 1–7 reporting the age (or weeks of age), body weight, and sex of the animals used in each study and summarize the conclusions derived from those data.
Moreover, the present review identified substantial concordance between nonclinical and clinical outcomes across many studies. These findings suggest that clinically relevant efficacy can often be predicted without requiring the use of aged animals, which appears inconsistent with the recommendations presented in Figure 2. The authors should therefore provide supporting evidence for these recommendations or revise the figure to align with the findings of the current review.

Reviewer #3

(Remarks to the Author)
We would like to thank the authors for their comprehensive amendments to the manuscript. We have no further requests.

Version 2:

Reviewer comments:

Reviewer #2

(Remarks to the Author)
I appreciate the authors’ response. However, my concern has not been fully addressed.

The revised Discussion provides a general rationale for considering age, sex, body composition, and comorbidities in OA models. However, it still does not explain the basis for the specific recommendations presented in Figure 2. These recommendations appear to extend beyond the evidence summarized in the present review.

Please either provide evidence supporting these specific recommendations or revise Figure 2 so that it presents them as general considerations rather than evidence-based recommendations. For example, instead of recommending specific ages,

sexes, or OA models, the figure could state:

“Consider age-appropriate animals”
“Include both sexes”
“Consider body composition and comorbidities”
“Select OA models according to the clinical phenotype of interest”

Such a revision would be more consistent with the evidence presented in the current review.

Open Access This Peer Review File is licensed under a Creative Commons Attribution 4.0 International License, which permits use, sharing, adaptation, distribution and reproduction in any medium or format, as long as you give appropriate credit to the original author(s) and the source, provide a link to the Creative Commons license, and indicate if changes were made.
In cases where reviewers are anonymous, credit should be given to 'Anonymous Referee' and the source.
The images or other third party material in this Peer Review File are included in the article’s Creative Commons license, unless indicated otherwise in a credit line to the material. If material is not included in the article’s Creative Commons license and your intended use is not permitted by statutory regulation or exceeds the permitted use, you will need to obtain permission directly from the copyright holder.
To view a copy of this license, visit https://creativecommons.org/licenses/by/4.0/

We thank the reviewers for their thoughtful and detailed revisions. Their valuable comments have been incorporated into the manuscript, which has been substantially improved as a result. Responses to the reviewers' comments are provided in blue for clarity.

Reviewers' comments:

Reviewer #1 (Remarks to the Author):

Stubelius and co-authors present a narrative review discussing the translational value of in vivo studies for development of treatments targeting pain in osteoarthritis. The topic is important and timely, and there are many interesting facts listed in this review. However, there are major concerns:

General Comments

1. The selection of the references included is not clearly articulated, both for the clinical trials as well as for the animal studies. For trials, it is stated that clinicaltrials.gov was searched, but the references included seem somewhat random. For example, for an important topic such as i.a. corticosteroids, two rather random references are provided (ref. 33 and 34). Better search criteria would be helpful. For the animal studies, the selection of models is not clear. Fig 1 specifies n=104 clinical studies with corresponding animal studies, but then many non-OA models are discussed, and it is not clear if those are all part of the n=104. The n=104 should be better described and inclusion criteria defined. For example, lines 221–230 discuss testing of SI-613 in a model of CIA in rats, while CIA is not an OA model but an RA model. A model using silver nitrate–induced OA is discussed, which is an atypical (and uncommon) OA model; it is more a subacute arthritis that is inflammatory in nature. For ref. 22, no pain results are discussed, only inflammation and cartilage (in a non-OA model).

The section describing the inclusion criteria for clinical trials and animal models has been revised for greater clarity (paragraph 2). External references related to the clinical trials discussed have been added to provide additional information. In addition, only preclinical studies specifically related to OA models were included. Studies addressing general joint diseases or other pain conditions, including the one suggested by the reviewer (Paragraph 2.2.1), as well as several others (Paragraph 2.2), have been excluded.

2. Further, there appear to be errors in the discussion/interpretation of certain references, e.g., ref. 27 is referred to as a carrageenan-induced paw edema (line 245) whereas in reality it is a murine DMM model; ref. 28 is discussed as a neuropathic

model and line 247 says “OA models were not available” while ref. 28 is a genuine OA model (ACLT) with solid pain outcomes.

- Reference 27 was incorrectly reported as a carrageenan-induced paw edema study. As correctly noted by the reviewer, this study instead used a destabilization of the medial meniscus (DMM) model in C57BL/6 mice to assess changes in cartilage structure. Since pain outcomes were not evaluated, the reference has been removed from the manuscript.
- Reference 28 refers to a study evaluating the efficacy of etoricoxib in an anterior cruciate ligament transection (ACLT) OA model in Wistar rats. The study assessed the compound’s effect on maintaining structural joint integrity and also evaluated secondary mechanical allodynia using the Von Frey test and paw weight distribution. The correct details of the study have now been integrated into the manuscript (Paragraph 2.2.1).

3. Translational relevance of certain pain outcomes is not always clear. For example, line 238, ref. 25, discusses heat sensitivity as an outcome in the MIA model, but is that translationally relevant? Have alterations in heat sensitivity been described in patients living with OA?

The reviewer highlighted an important aspect of preclinical research: the translational relevance of the outcome measures, in this case, heat sensitivity. Although OA is primarily characterized by mechanical pain, a subset of patients also exhibits altered thermal pain sensitivity, including increased sensitivity to heat. This phenomenon is thought to reflect peripheral and/or central sensitization mechanisms that amplify nociceptive processing beyond joint-specific mechanical loading. In the case of peripheral sensitization, joint inflammation and tissue damage increase nociceptor excitability. Moreover, central sensitization involves progressive amplification of pain signals within the spinal cord and brain, leading to exaggerated responses to normally non-painful or mildly warm stimuli.

Batchelor, Vicky, et al. "Peripheral neuronal sensitization and neurovascular remodelling in osteoarthritis pain." *Nature Reviews Rheumatology* 21.9 (2025): 526-545.

Dimitroulas, Theodoros, et al. "Neuropathic pain in osteoarthritis: a review of pathophysiological mechanisms and implications for treatment." *Seminars in arthritis and rheumatism*. Vol. 44. No. 2. WB Saunders, 2014.

Eitner, Annett, Gunther O. Hofmann, and Hans-Georg Schaible. "Mechanisms of osteoarthritic pain. Studies in humans and experimental models." *Frontiers in molecular neuroscience* 10 (2017): 349.

4. Frequently, specificity is lacking. As the subject of the review is the importance of OA

model choice, it is not appropriate to omit details around the model, such as Line 251, "In a rabbit OA model, celecoxib…". Likewise, the details of pain evaluation in all mentioned clinical and preclinical must be described thoroughly. For instance, lines 409-411: "where the preclinical data suggested a synergistic mechanism of action." Also lines 447-449. Please check the review for specificity.

We thank the reviewer for the suggestion. Appropriate details have now been added for each preclinical model mentioned and organized in a table. Changes were made in the entire paragraph 2.2.

5. There are many unfinished and unsupported statements. For instance, statements regarding companion animals are vague and unreferenced. These points need to made clear with concrete evidence and it is suggested to only discuss in section 3. As another example, specific points related to the benefit of using outbred mice should be reviewed from reference 101 and should be presented (Line 526).

The statement regarding the companion animals and the advantage of outbred animals in pain research has been elaborated more in section 3.

6. The illustrative figures (2 and 3) are unclear and lacking relevancy. As the theme of the review is whether preclinical trials relate to clinical successes/failures, the figures/tables should clearly synthesize and present this knowledge. For instance, this may be achieved with a main table that clearly lists the details of the preclinical model used (e.g. DMM, preclinical outcome, clinical trial details, clinical trial outcomes (WOMAC improvement/no sig. dif.).

As suggested by the reviewer, figure 2 has now been removed, and tables reporting details about clinical and preclinical data were organized in tables.

7. The authors should synthesize and report their findings regarding best preclinical pain evaluation method. For instance, might it be said that tactile allodynia led to less instances of predicting clinical trial success, as 3/3 studies discussed did not show significance in clinical trials compared to placebo? Points such as these should be discussed in Section 3. A synthesized figure or table as suggested above would assist in the saliency of these claims.

Summary of the comparison between clinical and preclinical trials was added for each pharmacological group reporting discrepancies between the studies. A comprehensive evaluation of the models, spontaneous pain versus evoked pain, timing at the beginning of the therapies, and for the pain assessment was presented in Section 3.

Specific Comments

Since the manuscript went through deep rearrangement and rewriting, all the comments below were addressed when still necessary.

1. Line 101, please specify ref 8 is about knee OA.
2. Line 152: the von Frey test assesses mechanical allodynia, not hyperalgesia.
3. Line 170: please provide a ref for this statement.
4. The DOI hyperlink for ref 23 leads nowhere.
5. Line 258: this was also true for diclofenac. The result is not interpreted. What does it mean if it only affected allodynia 3 days after induction? How does this relate to the clinical trials?
6. Line 294: here again, there's insufficient information provided around the model, and the results are not interpreted with respect to translational value.
7. Ref 42 is not correct
8. Statement in line 312 is missing a reference
9. Line 321: define analgesic activity.
10. Ref 60: this was hip OA- please discuss and provide details on the analgesic activity and what it means.
11. Line 342: "in contrast" is not appropriate here. Mechanical hyperalgesia cannot be contrasted to the WOMAC. Also, ref. 61 also measured allodynia.
12. Line 357: please provide refs. for the CNTX-4975-05 discussion
13. Grammar mistakes, including incomplete sentences should be fixed. Line 80: "Especially pain remains a major translational hurdle". Line 507, spelling in Figure 3.
14. Line 223-224: The treatment showed chondroprotective effects relative to what? Vehicle or Diclofenac?
15. Figure captions need more descriptions and abbreviations.
16. Spelling line 7: "on behalf"
17. Line 152: von Frey – no capital in "von".

Reviewer #2 (Remarks to the Author):

This review comprehensively summarizes, from the standpoint of the working group to which the authors belong, the limitations of current animal models in the development of novel analgesics targeting osteoarthritis (OA) pain. In particular, the collection of clinical trial data from ClinicalTrials.gov and the alignment of these data with findings from animal studies are valuable contributions.

However, the current format of data presentation and the comparison framework combine efficacy and safety in a way that makes it difficult for readers to grasp the overall picture.

The authors selected clinical trials and provided them as a supplementary table, but the results should be presented in a format that facilitates reader understanding—for example, a summary table compiling the investigated items (drug, route of administration, inclusion criteria, evaluation period, primary outcomes, etc.).
A summary of the comparison and discrepancies between preclinical and clinical trials is now present for each pharmaceutical group. The details are now presented in a table format to facilitate comparison.

Although the manuscript states that the 104 selected trials are those for which corresponding animal model data exist, these results are not shown. A summary table of the surveyed animal model data should also be created and presented.

The manuscript underwent extensive revision, and the first section now includes updated and expanded information about the data collection procedures. A supplementary table was created to report the clinical trials excluded.

In Section 2.2 ("Case studies"), the authors contrast results from clinical trials with those from animal models and describe their differences in a descriptive manner. To enhance reader comprehension, the corresponding clinical and nonclinical results should be organized into a comparative table. This table should specify which animal models were used (e.g., MIA model, rat strain, age, body weight, sex) and the methods of pain assessment (e.g., weight bearing, von Frey), together with the corresponding clinical trial outcomes.
We agree with the reviewer and comprehensive tables were generated to summarize important details for clinical and preclinical studies and facilitate comparison.

The discussion focuses primarily on the limitations of OA pain animal models. However, anti-NGF antibodies have demonstrated efficacy in both animal models and clinical settings, indicating that the current animal models can evaluate efficacy at least to some extent. Conversely, TRPV1 antagonists show efficacy for postoperative pain in animal models, whereas their clinical efficacy for postoperative dental pain is reported to be limited. This suggests that the issue is not only limitations in OA models but also potential species differences in the function of the drug target itself in pain mechanisms. The authors should discuss species differences in target biology related to pain, supported by relevant literature.

We thank the reviewer for this comment, which allowed us to address an important aspect of translational research: species-specific differences in pain mechanisms (added to the discussion). The fundamental mechanisms of pain perception, such as nociception, are evolutionarily conserved between rodents and humans. However, important differences exist in the central processing and modulation of pain signals. In particular, the organization of ascending pain pathways differs between rodents and humans, as do descending modulatory pathways, including differences in sensitivity to neurotransmitters such as serotonin. These differences may limit the predictive value of rodent models for therapies targeting central pain mechanisms. Moreover, species-specific differences have also been described for the TRPV1 channel. This receptor is expressed in approximately 32% of sensory neurons in humans, compared with about 78% of somatosensory neurons in rodents. Such differences may partly explain why TRPV1 modulators can produce distinct effects in rodents and humans.

The discrepancy between expected effects from animal models and insufficient efficacy in clinical pain trials is not limited to OA pain; it is also observed in the development of drugs for neuropathic pain and other pain conditions. This stems fundamentally from the subjectivity of “pain” in clinical settings, which cannot be directly assessed in animals. While the authors conclude that future work should focus on improving OA pain animal models, they should also discuss the broader issue of translational challenges in pain itself, along with possible solutions (e.g., brain imaging, experimental human model).
We agree with the reviewer that a broader discussion of the translational challenges in pain research is both important and highly relevant. In the final section of the manuscript, we discuss several approaches to improve the translational value of laboratory animal research. However, due to space limitations, we have focused the review on its primary objective: evaluating the translational validity of in vivo animal research in OA pain research.

Reviewer #3 (Remarks to the Author):

Summary of the manuscript
This review article aims to examine the translational gap underlying the failure to develop effective pain-targeting therapies for clinical osteoarthritis (OA). The authors introduce the scale of the problem, highlighting some of the difficulties in recapitulating complex multifaceted diseases in simplified animal models, and then state the aim of critically retracing the path from clinical observations back to animal models, and

proposing strategies to enhance translational relevance. Mechanisms of OA in humans and animals, and differences in methods of assessing of pain are discussed.

The overall approach of the review is to identify completed phase 2 & 3 clinical trials with OA pain as the primary end point, and to compare the findings to corresponding published preclinical studies using the same agents. The aim is to identify potential reasons underlying clinical failure of some analgesics. Clinical studies are categorised based on the pharmacological intervention (NSAIDs, corticosteroids, opioids, antidepressants and anticonvulsants, TRPV1 modulators, biologic drugs, and other small molecule inhibitors), and compared to the preclinical literature. The authors then make suggestions for refining experimental design to improve translatability, concluding that future research should focus on tailoring animal models to specific patient populations, rather than using general disease models.

Overall impression of the work
This is an important area for review, particularly given the recent announcements from the UK government and FDA of policies aiming to phase out and replace animals in scientific research. A review of how well animal models align with clinical studies in OA is therefore timely. It is however surprising that the role of novel non-animal methodologies to address the translational gap is not considered (see comment 3 below).
The approach of tracing clinical trials back to the preclinical literature to identify the reasons for differences in effectiveness of treatments is interesting, and the authors comprehensively collate completed trials. However, they provide less detailed coverage of preclinical studies and the level of citations is weak in places, in addition the two are not always well linked.

We thank the reviewer for acknowledging the importance of the review in this field. The manuscript went through extensive revision: data collection with inclusion and exclusion criteria were better described and details of the studies are presented in a table format with the main text including a short description of the studies and a summary about the discrepancies in the study design between the preclinical and clinical studies for each pharmacological group examined.

The overall aim of this review is unclear as it is variously stated as “critically examining how current in vivo models align – or fail to align – with the clinical reality of OA pain in humans” (page 4 line 86) and “to assess the design and outcomes of clinical trials, with a specific emphasis on discrepancies relative to preclinical animal data” (page 8, line 173)- it si not clear what they mean by ‘discrepancies relative to preclinical animal data’.

A consistent overall aim would help to focus the manuscript, which currently does not meet either of these statements, and there are several other areas for improvement.

The aim of the study has been elaborated in a clearer form in the abstract and in the introduction section.

Specific comments and recommendations

1. Although the focus is on analgesic development for OA pain, the review would benefit from a wider perspective of other chronic pain states, such as neuropathic pain states and relevant experimental models. This will ensure that the audience is aware that there are similar translational gaps for other chronic pain states. They could then highlight some key common areas relevant to the underlying basis for the translational gap. Areas that could be considered for inclusion: heterogeneity of clinical pain conditions, the complexity and diversity of underlying pathophysiological mechanisms, and the unreliability of some preclinical pain models. Lack of predictive validity (discussed in Yekkirala et al. 2017); discordance between preclinical and clinical testing - heterogeneity in clinical populations vs homogeneous animal subjects, types of pain mechanisms studied (discussed in Yang et al., 2025); poor alignment of clinical and preclinical endpoints, need more translational endpoints, greater importance of spontaneous (non-evoked) pain behaviour (discussed in Fischer et al 2021, Huerta et al., 2025).

We agree with the reviewer that translational challenges are highly relevant to the pain research field. Although a broader overview of these challenges would be of interest, space limitations led us to focus specifically on pain in osteoarthritis. Nevertheless, in the final section of the manuscript, we discuss several key translational aspects relevant to this topic, including population heterogeneity, evoked versus spontaneous pain, as well as the selection of endpoints and timing of treatment interventions.

2. Description & discussion of animal models – The authors state that a critical issue with animal models is that they do not mimic the full complexity of OA in humans (line 79). Whilst this is undoubtedly true, it is also well-recognised that OA is a heterogeneous clinical diagnosis which likely represents a collection of diseases, which can be stratified into different phenotypic presentations with varying underlying pathologies. The majority of induced models of arthritis aim to mimic key aspects of the disease (e.g. OA-like pain) or specific interactions (e.g. co-morbid anxiety an OA-like pain), rather than the disease state as a whole, which is in line with the authors own recommendations (Figure 3). There are numerous examples of back-translation of clinical phenotypes in the literature, and these should be cited.

The authors mention the potential utility of OA in companion animals (line 126, line

589), but do not elaborate or provide supporting references. These should be included, and the suggestion of greater involvement of, and consultation with, veterinarians expanded upon. It would also be worth including some of the extensive literature on spontaneous OA in Dunkin-Hartley guinea pigs (Jimenez, Glasson et al. 1997) as a more ethologically relevant model, and the potential for opportunistic preclinical studies of OA in larger animals such as pigs, sheep, goats, & horses (Stewart, Gilbert et al. 2024) as a means to circumvent issues of cost in ageing research animals up for clinical relevance to the chronic pain seen clinically (lines 532-537).

In the discussion section of the review, we also highlight the importance and potential advantages of studies based on veterinary data and on animals that spontaneously develop osteoarthritis.

The statement that the rodent MIA model of OA as not reproducing the structural joint changes of OA (line 146) is not supported by the literature. Numerous studies have identified clinically relevant structural changes in the MIA model, including synovium, cartilage, and subchondral bone, and this should be acknowledged. Similarly, the authors state that to model the heterogeneity of the human pain experience, outbred strains of rodents should be used, rather than inbred C57BL/6 mice. This ignores the fact that many studies are conducted using outbred rat strains such as Sprague Dawley and Wistar, which are notably genetically heterogeneous (Gileta, Fitzpatrick et al. 2022)

We agree with the reviewer that the contribution of outbred rat strains was insufficiently addressed. This has now been corrected in the final section of the manuscript. In addition, the discussion of the relevance and limitations of the MIA model has been expanded.

In later descriptions of studies, key details of the models, strain and sex of animals, and endpoints measured are lacking. Given the aim of the review to investigate the relevance of preclinical models to clinical populations and to identify reasons for the translational gap, it is important to describe how each model works, and what aspect of OA it is modelling. For example, when the effects of diclofenac are described in three models of arthritis (page 10, lines 221-230), it is not clear how arthritis is induced in the silver nitrate model in male Sprague Dawley rats, and no details are provided on what constitutes the rabbit model of arthritis (line 229) or which sexes were involved. This is also true of the rabbit OA model in which celecoxib was tested (line 251) and strain of rats used for testing melocoxicam (Wistar, line 236). Often multiple models are discussed without clearly delineating the differences (e.g. ACLT, pMMx & MIA in rats, line 469). Section 1.3 could be expanded to include brief descriptions of these models, or they could be introduced individually when first described.

We thank the reviewer for this valuable suggestion. A more detailed description of the OA models has now been added, and studies relying on OA models that are not widely accepted in the field have been removed.

3. Description of differences in pain assessment (Page 7, line 149-157). The review of the assessment of pain behaviour would benefit from greater detail and appropriate referencing. The excellent review by Zaki (2025) provides considerable detail on selection of model, consideration of outcome measures and relevant factors to consider when designing a preclinical study. Greater referencing of this review would be beneficial. This section could be developed to highlight opportunities to provide more translatable measures of pain in OA (Tappe-Theodor, King et al. 2019). For example, quantitative sensory testing (QST, line 169) is analogous to assessment of withdrawal thresholds in preclinical models & provides an opportunity to more closely translate findings between the two. This section could be developed by linking to studies that have used temporal summation of QST responses to identify neuropathic pain components in OA patients, with predictive power for determining incidence of pain persistence following total joint replacement. Whilst preclinical studies cannot replicate the subjective measures of pain intensity reported in the clinic, use of paradigms such as conditioned place preference, and studies that have attempted to back-translate conditioned pain modulation (CPM) testing in pain patients to preclinical studies should be mentioned.

We thank the reviewer for the meaningful suggestion. Considerations on a more translatable measure of pain in OA have been added in the last section of the manuscript. The conditioned place preference has been added as a suggested paradigm for preclinical studies.

4. The overall level of detail (and supporting references) will aid comprehension and improve the quality of the review. For example- page 5, line 120-123- clearer description of the differences and provide the supporting evidence. Page 5/ 6 (line 125-129) the utility of companion animals- inclusion of relevant citation required. In addition, any limitations (study design, controls, objective endpoints) in the study design should be included to ensure balance in the narrative (see above point).

The manuscript went through deep modification to add more information and details important for the comprehension of the differences and discrepancies in the studies.

Page 6, (line 144-147): The discussion of the MIA model requires citations supporting the statement that it doesn’t reproduce structural changes seen in OA (see point 2). There are multiple papers from multiple groups reporting changes to cartilage, bone and synovium and therefore it would be useful for the authors to be clear which structural

joint changes are not reproduced whilst providing balance in the reporting of those features that are exhibited and clinically relevant. The review cited Zaki et al. 2025 does state that the MIA model may not recapitulate the pathophysiology of OA or the molecular changes seen in humans. The second review cited here (Rosi et al 2022, which is authored by one of the co-authors herein) doesn't appear to mention the MIA model in any detail and therefore isn't ideal for this citation. Missing references – some statements are unsupported by references, e.g. studies in the DMM model which have predictive power for the joint safety of opioids & anti-NGF treatments, lines 541-5.a

As mentioned earlier, the MIA model has been described with better details in the introduction and in the last section of the manuscript. Inclusion of the study cases was better explained in the Data collection paragraph.

5. Alternative methodologies to animal models - Given the recent announcements from the UK government (https://www.gov.uk/government/publications/replacing-animals-in-science-strategy) and FDA (https://www.fda.gov/news-events/press-announcements/fda-announces-plan-phase-out-animal-testing-requirement-monoclonal-antibodies-and-other-drugs) of policies aiming to replace animals in scientific research, it is important for reviews in this area to consider the contribution of new approach methodologies, such as 3D multi cell-type culture systems, & organs on chips, particularly those produced from relevant patient iPSCs, or computation models in replacing some aspects of animal models. E.g. recent commentary in nature (Kwon 2026). Whilst this is not the major focus of the review, discussion of how this applies to complex multi-system/level diseases such as OA and recent studies that have attempted this (Salgado, Jordan et al. 2021, Ummarino, Pensado-López et al. 2023, Marrero - Berrios, Salter et al. 2024) would be a welcome addition.
We agree with the reviewer that alternative methodologies to animal models represent an important and timely topic. We briefly mention the use of New Approach Methodologies in translational research in the introductory section. However, this is a complex area that extends beyond the primary scope of the present review. To maintain focus and remain within the space limitations, we prioritized the inclusion of information directly related to the translational validity of animal models in OA pain research.

6. Effect sizes – In order to compare efficacy of drugs between clinical & preclinical studies, it is necessary to give an indication of the effect sizes reported, rather than just stating that, for example, a treatment reduces pain. This can be challenging when measurements of pain are not equivalent (e.g. self-reported pain in humans and withdrawal thresholds in rodents), but an indication of relative efficacy would still be useful. This will also aid in comparing between different classes of compounds.

We agree with the reviewer that the effect size can provide valuable information regarding the degree of improvement following a specific treatment. However, in our context, direct comparisons of effect sizes are not informative. The compounds belong to different classes and rely on distinct pain measurement tools, making direct comparisons across studies unfeasible. This limitation is even more pronounced when comparing preclinical and clinical trials, given that pain assessments are not equivalent, as the reviewer rightly points out. Moreover, comparisons among preclinical studies themselves are also not interpretable due to substantial heterogeneity in experimental designs, including differences in species, strains, disease models, treatment timing, and pain assessment methods.

7. The contribution of neuropathic pain mechanisms (Page 5, lines104-107). This requires some more consideration. The sentence line 104-106 relating to stretched or compressed articular nerves requires a reference and the authors need to be clear on what is meant by the statement ‘emergence of symptoms recognised as neuropathic pain’. The reference cited (Hoffman et al. 2025) does not discuss neuropathic pain, it does refer to neuroplasticity, but this can be a mechanism contributing to nociceptive and neuropathic pain. The IASP definition of neuropathic pain provides a clear framework to support distinctions between possible underlying mechanisms.
The paragraph mentioned has been edited for clarity.

8. Relationship between pain and anxiety and impact on treatment outcomes. Page 5 (lines 116-119) discusses comorbidities and influences on pain perception, this could be expanded to discuss anxiety and the impact of anxiety on chronic pain and treatment responses. This is relevant to the phenotyping of OA pain, mapping to pain mechanisms which informs treatment selection (Figure 3 -tailored treatment).
As the reviewer noted, anxiety significantly influences the perception and experience of pain. Considerations regarding pain-related anxiety, including its underlying mechanisms and clinical relevance, have been incorporated into the first section of the manuscript.

9. Consistency between findings (clinical versus pre-clinical). The authors correctly identify that there is consistency for NSAIDs, this is also the case for opioids-but this isn’t highlighted in the review and could be expanded upon. There is also consistency for anti-NGF treatments. Page 17 (lines 386-391) mentions one study, there are multiple other examples where anti-NGF treatments were shown to have beneficial effects in rodent models of OA pain (PMID: 29100502, PMID: 27208420, PMID: 25677108, PMID: 25966657, PMID: 30862648) - the rationale for the study selected isn’t clear, and greater balance would be provided by a broader group of citations.

More details on the study selection process have now been provided, and the application of the revised selection criteria has increased the number of included preclinical studies.

10. Conclusions – the concluding statement reads more as a list of limitations and doesn't summarise the overall findings and recommendations of the review, which are not entirely clear. From Figure 3, it appears that a major suggestion is to back-translate clinical phenotypes to more specifically model patient populations, particularly the use of female rodents with comorbid obesity and/or diabetes to achieve this. This proposal would be strengthened by the inclusion of studies which have already undertaken this type of more complex modelling. The use of a combination of models, including those that mimic different facets of clinical OA in multiple species, would appear to be a fitting conclusion.
In response to the reviewer's suggestions, we have refined the concluding statement to present more concise recommendations. Additionally, Figure 3 has been revised and embedded within a more comprehensive infographic to better illustrate the key messages.

The conclusion discusses both human endotypes and clinical phenotypes and importantly in the context of this review the 'limited information on endotype specific features'. It would be beneficial if the authors were clearer on the meaning of terms endotype and phenotype in the context of OA pain mechanisms, as they appear to be inter-changeable. Our reading of this section is that lack of 'consensus in the clinical phenotypes' still needs to be overcome so that it is clear which clinical phenotypes need to be modelled to address the translational gap in the development of new analgesics for OA pain. This may also be a fitting conclusion.

Formatting issues & minor suggestions

Formatting issues have been corrected. We have also adopted the reviewer's suggestions, including a new title and an updated Figure 2 (before figure 3).

Article title – the current phrasing is a little unclear, and does not fully represent the aim of the review, suggest an alternative along the lines of:
Assessing the translational value of animal models in developing pain-targeting therapies for clinical osteoarthritis
Line 154 – the open field test is not considered to be ideal for assessing spontaneous locomotor activity as it can be considered anxiogenic, and this may interfere with interpretation in conditions such as OA where anxiety and physical disability are comorbid. Locomotor activity measurement systems, or those that combine observation

of naturalistic behaviours and gait parameters would be preferable.

Figures - figure 2 is titled as a graph, but would be better described as an infographic. It has potential to be useful to the reader, but a comprehensive legend should be provided containing all the information required to understand it. This includes definitions of acronyms and abbreviations, and a narrative description of what the authors are trying to explain. It is not currently clear how the structural phenotypes listed at the top relate to the pain mechanisms in the bottom 2 panels, and the sites of actions of pharmacological treatments are incomplete or unclear. Figure 3 could be improved by refinement of the text on the graphic – it is not clear what the obesity OA model is, or how mismatch structural changes - pain intensity, women are more represented, high BMI of patients, and chronic pain feeds back into the preclinical models (grey arrow, lower right-hand side) – and a comprehensive figure legend.

Acronyms & abbreviations should be defined at first usage, e.g. CGRP in line 391 rather than 443.

Formatting – italics for latin (e.g. in vivo, line 547, 599)

References – numerical citations in the text should appear before full stops rather than after.

One of the supplementary tables (8037_0_supp_184648_t7qckt) does not have a title, explanation, or table number

References

Fischer AS, Lanigan MT, Upton N, Lione LA (2021) Preclinical neuropathic pain assessment: the importance of translatability and bidirectional research. Frontiers in Pharmacology 11, 614990

Gileta, A. F., C. J. Fitzpatrick, A. S. Chitre, C. L. St. Pierre, E. V. Joyce, R. J. Maguire, A. M. McLeod, N. M. Gonzales, A. E. Williams, J. D. Morrow, T. E. Robinson, S. B. Flagel and A. A. Palmer (2022). "Genetic characterization of outbred Sprague Dawley rats and utility for genome-wide association studies." PLOS Genetics 18(5): e1010234.

Huerta MA, Roza P, Cisneros E, Alique M, Roza C (2025). Enhancing clinical translation of analgesics for neuropathic pain: a systematic review and metaanlysis on the role of non-evoked pain assessment in preclinical trials. Pain 166(11) 2473-2486.

Jimenez, P. A., S. S. Glasson, O. V. Trubetskoy and H. B. Haimes (1997). "Spontaneous osteoarthritis in Dunkin Hartley guinea pigs: histologic, radiologic, and biochemical changes." Lab Anim Sci 47(6): 598-601.

Kwon, D. (2026). "The age of animal experiments is waning. Where will science go next?" Nature 650(8103): 812-814.

Marrero - Berrios, I., S. E. Salter, R. Hirday, C. P. Rabolli, A. Tan, C. T. Hung, R. S. Schloss and M. L. Yarmush (2024). "In vitro inflammatory multi-cellular model of osteoarthritis." Osteoarthritis and Cartilage Open 6(1): 100432.

Salgado, C., O. Jordan and E. Allémann (2021) "Osteoarthritis In Vitro Models: Applications and Implications in Development of Intra-Articular Drug Delivery Systems."

Pharmaceutics 13, 60 DOI: 10.3390/pharmaceutics13010060.
Stewart, H. L., D. Gilbert, D. Stefanovski, Z. Garman, M. B. Albro, M. Bais, M. W. Grinstaff, B. D. Snyder and T. P. Schaer (2024). "A missed opportunity: A scoping review of the effect of sex and age on osteoarthritis using large animal models." Osteoarthritis and Cartilage 32(5): 501-513.
Tappe-Theodor, A., T. King and M. M. Morgan (2019). "Pros and Cons of Clinically Relevant Methods to Assess Pain in Rodents." Neuroscience & Biobehavioral Reviews 100: 335-343.
Ummarino, A., A. Pensado-López, R. Migliore, L. Alcaide-Ruggiero, N. Calà, M. Caputo, F. M. Gambaro, C. Anfray, F. L. Ronzoni, E. Kon, P. Allavena and F. Torres Andón (2023). "An in vitro model for osteoarthritis using long-cultured inflammatory human macrophages repeatedly stimulated with TLR agonists." European Journal of Immunology 53(12): 2350507.
Yang J, Xie, Y-F, Smith R, Ratte, S, Prescott, SA (2025) Discordance between preclinical and clinical testing of NaV1.7-selective inhibitors for pain. Pain 166 (3) 481-501.
Yekkirala AS, Roberson DP, Bean BP, Woolf CJ (2017) Breaking Barriers to novel analgesic drug development. Nature Rev Drug Discov 16 (8) 545.

Vicky Chapman and Stephen Woodhams, Arthritis UK Pain Centre, University of Nottingham

Dear reviewer,

We sincerely thank you for the further careful and constructive evaluation of our manuscript. We have carefully considered all of the comments raised during this second round of review and have revised the manuscript accordingly. We provide below a point-by-point response to each comment and indicate the corresponding changes made in the revised manuscript.

Comment 1 (Discussion, Lines 604–605)
In the Discussion (Lines 604–605), the authors state that “While the MIA model is valuable for studying acute inflammatory processes, it does not mirror the slow and progressive trajectory of human OA.” However, many of the findings presented in this review demonstrate substantial concordance between the clinical outcomes and the results obtained in the MIA model. Therefore, the data summarized in this manuscript do not appear to support this statement.
Furthermore, Reference 26 also indicates that the MIA model reflects key aspects of clinical OA, which is inconsistent with the above assertion. The authors should provide clear evidence and justification supporting this statement or revise the discussion accordingly.

We thank the reviewer for noting the inconsistency between the data summary and the statement in the Discussion. The MIA model does not fully recapitulate the temporal progression of joint damage observed in human OA. In fact, monoiodoacetate is an inhibitor of glyceraldehyde-3-phosphate dehydrogenase that disrupts cellular glycolysis. When injected into the joint space, it induces chondrocyte cell death and a high degree of inflammation. Although this model is convenient and relatively straightforward to use, the rapid progression of joint damage and the lack of differentiation between the medial and lateral compartments do not fully recapitulate the progression of human disease, as observed in surgical animal models (Aüllo-Rasser, G., Dousset, E., Roffino, S. et al. Early-stage knee OA induced by MIA and MMT compared in the murine model via histological and topographical approaches. Sci Rep ***10****, 15430 (2020). https://doi.org/10.1038/s41598-020-72350-7). However, this model has been shown to be a valid and widely used model for assessing the efficacy of pain-modulating interventions. We have therefore revised the statement in the Discussion to reflect this concept more accurately.*

Comment 2 (Discussion, Lines 611–614)
In the Discussion (Lines 611–614), the authors state:
“Consistent with this, the MIA model shows good concordance with clinical outcomes for therapies targeting inflammation, such as NSAIDs, opioids and corticosteroids, particularly in early stages of the disease. However, its predictive value declines in later disease stages, when chronic pain mechanisms dominate.”
However, as described by the authors themselves in the opioid section, opioids are generally used for “moderate-to-severe symptoms insufficiently controlled by first-line therapies,” indicating that opioids primarily target chronic pain in more advanced stages of OA rather than inflammation.
Therefore, including opioids as examples of anti-inflammatory therapies is inappropriate, and the statement regarding the predictive limitations of the MIA model in later disease stages is

not supported by the findings presented in this review. In fact, the current analysis shows that opioids demonstrate efficacy in the MIA model that is largely consistent with their clinical efficacy. Accordingly, this section should be revised.

Although the primary therapeutic target of opioids is pain modulation, their immunomodulatory effects have also been demonstrated (Schäfer, M., & Zöllner, C. (2013). Opioids and Inflammatory Pain. In: Gebhart, G.F., Schmidt, R.F. (eds) Encyclopedia of Pain. Springer, Berlin, Heidelberg. https://doi.org/10.1007/978-3-642-28753-4_2977; Sacerdote, P., Franchi, S., & Panerai, A. E. (2012). Non-analgesic effects of opioids: mechanisms and potential clinical relevance of opioid-induced immunodepression. Current Pharmaceutical Design, 18(37), 6034–6042). However, these effects are heterogeneous and highly context-dependent. We therefore acknowledge that referring to an anti-inflammatory effect of opioids may be misleading in the context of the present analysis. Accordingly, we have revised the statement to clarify this point.

Comment 3 (Figure 2: MIA Model)

In Figure 2, cartilage degeneration in the MIA model is indicated as occurring at 28 days. However, the basis for this specific time point is not provided anywhere in the manuscript. Since the purpose of Figure 2 is to summarize the findings of the present review, all information presented in the figure should be directly supported by the data collected in this study. The authors should therefore add a new column to Tables 1–7 indicating whether cartilage degeneration was observed in each animal model reviewed, and summarize the findings derived from those data.

In addition, the timing of allodynia and hyperalgesia should be revised. Based on the literature reviewed, it would be more appropriate to indicate allodynia as occurring 'from days 5–10 onward' and hyperalgesia 'from day 3 onward'.

We thank the reviewer for this comment. The top-right panel is intended to provide an overview of the current knowledge regarding the models used and does not directly refer to the data summary. Rather, it is intended to support the Discussion and Conclusions sections. In contrast, Tables 1–7 specifically support the data summary, which focuses on pain modulation; therefore, we believe that adding column on cartilage damage would not be appropriate within the scope of these tables. The statement regarding the 28-week duration of the MIA model is supported by Reference 26, as indicated in the main text.

Following the reviewer's suggestion, we have also added the term "onward" to the indicated timing to further clarify the temporal aspect.

Comment 4 (Figure 2: Learn from the Clinical Experience)

In the "Learn from the Clinical Experience" section of Figure 2, the authors suggest that a 3 month old ACLT model is appropriate for modeling OA in males and that a 7 month old animal model is appropriate for females. However, the manuscript does not provide any supporting evidence for these recommendations.

As Figure 2 should reflect the findings of the present review, the authors should add additional columns to Tables 1–7 reporting the age (or weeks of age), body weight, and sex of the animals used in each study and summarize the conclusions derived from those data. Moreover, the present review identified substantial concordance between nonclinical and clinical outcomes across many studies. These findings suggest that clinically relevant efficacy can often be predicted without requiring the use of aged animals, which appears inconsistent with the recommendations presented in Figure 2. The authors should therefore provide supporting evidence for these recommendations or revise the figure to align with the findings of the current review.

The Discussion and Figure 2 are intended to highlight the importance of considering sex, age, and body weight as essential variables when designing and interpreting OA models. Although the summarized data, with the exception of senolytics, generally show good concordance between findings in humans and those observed in young animals, this summary should be interpreted with caution. In particular, negative or inconclusive results may be less likely to be published and, consequently, may not be captured in the available literature or included in our analysis. We believe that this potential publication bias should not be overlooked when drawing general conclusions and providing recommendations for the development and selection of OA models.

Moreover, refinement of animal models is particularly important in the context of better defining clinically relevant OA phenotypes, which may ultimately contribute to the development of more effective and targeted strategies for modulating OA pain.

Reviewers' comments:

Reviewer #2 (Remarks to the Author):

I appreciate the authors' response. However, my concern has not been fully addressed. The revised Discussion provides a general rationale for considering age, sex, body composition, and comorbidities in OA models. However, it still does not explain the basis for the specific recommendations presented in Figure 2. These recommendations appear to extend beyond the evidence summarized in the present review.

Please either provide evidence supporting these specific recommendations or revise Figure 2 so that it presents them as general considerations rather than evidence-based recommendations. For example, instead of recommending specific ages, sexes, or OA models, the figure could state:

"Consider age-appropriate animals"

"Include both sexes"

"Consider body composition and comorbidities"

"Select OA models according to the clinical phenotype of interest"

Such a revision would be more consistent with the evidence presented in the current review.

Response: We thank Reviewer #2 for his time and for the constructive suggestion. We understand the concern that the specific recommendations presented in Figure 2 may appear to extend beyond the evidence directly summarized in the present review.

In response, we have revised Figure 2 to present these factors as general considerations for the design and selection of preclinical osteoarthritis models rather than as specific evidence-based recommendations. Specifically, the wording has been modified to emphasize consideration of age-appropriate animals, inclusion of both sexes where appropriate, consideration of body composition and relevant comorbidities, and selection of OA models according to the clinical phenotype or research question of interest.
