# Supplementary material for: Assessing the translational value of animal models in developing pain-targeting therapies for clinical osteoarthritis
Source: Commun Med (Lond). 2026 Oct 8;6:522. doi: 10.1038/s43856-026-01926-7 (PMC13649651; doi:10.1038/s43856-026-01926-7)
Supplement: Supplementary file 2 [file 43856_2026_1926_MOESM2_ESM.pdf]

Supplementary Table 1. **Clinical studies included in the review.** The table provides an overview of the clinical trials identified through a search of ClinicalTrials.gov. Homeopathic interventions, herbal therapies, and studies outside the scope of this review were excluded. Further exclusions, as specified in the table, were applied to studies lacking a corresponding preclinical animal study, studies for which results or data analyses were not publicly available, and studies that compared the efficacy of different drug formulations without including a control or vehicle group.

	NCT ID<br>Study period	Name of the clinical trial	Exclusion reason
NSAID	NCT00092768<br>2004-03/2005-02	A Study of MK0663 and an Approved Drug in the Treatment of Osteoarthritis (0663-076)	Included
	NCT00092781<br>2004-03/2005-02	A Study of MK0663 and an Approved Drug in the Treatment of Osteoarthritis (0663-077)	Included
	NCT01461369<br>2011-10/2012-10	Study of Diclofenac Capsules to Treat Osteoarthritis Pain	Included
	NCT01554163<br>2012-03/2013-03	Safety and Efficacy of Etoricoxib 30 mg Versus Celecoxib 200 mg in Korean Participants With Osteoarthritis	Included
	NCT01787188<br>2013-03/2013-10	Study of Meloxicam Capsules to Treat Osteoarthritis Pain	Included
	NCT01980940<br>2013-12/2014-11	The Single Dose Pharmacokinetics of Two and Proof of Efficacy of One New Etoricoxib Gel Formulation in Participants With Osteoarthritis (MK-0663-168)	Included
	NCT02311881<br>2015-01/2016-02	A 12-Week Efficacy Study of Paracetamol 1000mg Sustained-release Tablets in Patients With Osteoarthritis	Included
	NCT03209362<br>2017-10/2018-11	SI-613 Study for Knee Osteoarthritis	Included
	NCT05007808<br>2021-01/2023-01	Efficacy and Safety of G001 in Patients With Osteoarthritis (OA) of the Knee	Included
	NCT00664560<br>2008-04/2009-01	Efficacy Study of PN400 (VIMOVO) Twice Daily and Celebrex Once Daily in Patients With Osteoarthritis	No relevant animal study identified

	NCT00665431 2008-04/2009-01	Efficacy Study of PN400 (VIMOVO) Twice Daily and Celebrex Once Daily in Patients With Osteoarthritis	No relevant animal study identified
	NCT01147458 2010-07/2011-02	A Study Of The Safety And Efficacy Of PF-04191834 In Patients With Osteoarthritis Of The Knee	No relevant animal study identified
	NCT01119898 2010-08/2011-02	Exploratory Study of PENNSAID Gel to Treat Symptoms of Knee Osteoarthritis	No relevant animal study identified
	NCT01341405 2011-04/2012-01	Study of CG100649 Versus Celecoxib in Osteoarthritis Patients	No relevant animal study identified
	NCT01765296 2013-03/2014-04	Phase III Study of CG100649 in Osteoarthritis Patients	No relevant animal study identified
	NCT01799213 2013-09/2018-06	Discontinuing NSAIDs in Veterans With Knee Osteoarthritis	No control/placebo comparison
	NCT01967550 2013-10/2014-05	Efficacy and Safety of Diclofenac DDEA 2.32 % in Patient Suffering From Knee Osteoarthritis (OA)	No data analysis reported
	NCT02067611 2014-02/2016-04	A Study to Evaluate the Efficacy and Safety of X0002 Spray in Subjects With Osteoarthritis	No data analysis reported
	NCT02121002 2014-04/2014-12	Study Comparing a Generic Diclofenac Sodium Topical Gel, 1% to Voltaren in the Treatment of Subjects With Osteoarthritis of the Knee	No data analysis reported
	NCT02311881 2015-01/2016-02	A 12-Week Efficacy Study of Paracetamol 1000mg Sustained-release Tablets in Patients With Osteoarthritis	No relevant animal study identified
	NCT02913521 2015-06/2016-06	Bioequivalence Study With Clinical Endpoint for Diclofenac Sodium Topical Gel 1%	No data analysis reported
	NCT02596451 2015-08/2016-03	To Study Generic Diclofenac Sodium Topical Gel 1% in the Treatment of Osteoarthritis of the Knee (Degenerative Joint Disease Presented as Joint Pain, Stiffness, Swelling and Restricted Motion).	No data analysis reported
	NCT03172780 2017-05/2017-12	A Bioequivalence Study Using Clinical Endpoint for Diclofenac Sodium Gel 1% in Osteoarthritis Knee*	No data analysis reported
	NCT03277066	A 4-week Study to Evaluate the Efficacy and Safety of HP-5000 in Subjects With Knee Osteoarthritis	Comparison between formulations

Supplementary table 1

	2017-09/2019-09		
	NCT03691844<br>2018-10/2019-07	AMZ001 for the Treatment of Knee Osteoarthritis Symptoms	No relevant animal study identified
**Corticosteroid**	NCT02116972<br>2014-04/2015-11	Study of FX006 vs Normal Saline in Patients With Osteoarthritis of the Knee	Included
	NCT02357459<br>2015-01/2016-01	Study of FX006 for the Treatment of Pain in Patients With Osteoarthritis of the Knee	Included
	NCT00521989<br>2007-08/2009-07	CRx-102 Osteoarthritis Multicenter Evaluation Trial	No relevant animal study identified
	NCT01487161<br>2012-06/2013-04	Study of FX006 in Patients With Osteoarthritis of the Knee	No control/placebo comparison
	NCT02468583<br>2015-02/2016-07	Proof of Concept Study Comparing FX006 to Kenalog®-40 in Patients With Post-Traumatic Osteoarthritis of the Knee	No control/placebo comparison
	NCT02688400<br>2016-05/2018-06	Effect of Diacerein vs Celecoxib on Symptoms and Structural Changes in Symptomatic Knee Osteoarthritis	No control/placebo comparison
	NCT03005873<br>2017-03/2018-07	Phase IIa, Randomized, Double Blinded, Placebo Controlled, Dose Finding Study for TLC599 in OA Patients	No relevant animal study identified
	NCT03191903<br>2017-05/2018-04	Study of Cingala™ for the Relief of Knee Osteoarthritis Compared to Triamcinolone Hexacetamide	No relevant animal study identified
	NCT03390036<br>2017-12/2018-07	Study of Cingal for the Relief of Knee Osteoarthritis Compared to Triamcinolone Hexacetamide at 39 Weeks Follow up	No relevant animal study identified
	NCT03793010<br>2018-12/2019-08	Study to Evaluate the Efficacy and Safety of FX006 in Patients With Hip Osteoarthritis	No data analysis reported
	NCT04160091<br>2019-11/2020-06	Study to Evaluate the Efficacy and Safety of FX006 in Patients With Glenohumeral Osteoarthritis or Shoulder Adhesive Capsulitis	No data analysis reported
	NCT04123561<br>2019-11/2022-02	Extended and Controlled Release Liposomal Formulated Dexamethasone for Chronic Knee OA Pain	No relevant animal study identified

	NCT04231318<br>2020-09/2022-05	Study of Cingal® and Triamcinolone Hexacetamide for the Relief of Knee Osteoarthritis Pain	No relevant animal study identified
	NCT04120402<br>2021-09/2023-06	Study to Evaluate the Efficacy and Safety of EP-104IAR in Patients With Osteoarthritis of the Knee	No relevant animal study identified
**Opioid**	NCT00950651<br>2002-03/2002-11	A Comparison of the Analgesic Efficacy and Safety of Once Daily Tramadol HCl / ContramidA® Tablets to Twice Daily Tramadol HCl (SR) for the Treatment of Osteoarthritis of the Knee	Included
	NCT00832416<br>2003-01/2003-08	A Four-Arm Study Comparing the Analgesic Efficacy and Safety of Tramadol Once a Day 100, 200 and 300 mg Versus Placebo for the Treatment of Pain Due to Osteoarthritis of the Knee	Included
	NCT00852917<br>2003-01/2003-08	A Four-Arm Study Comparing the Analgesic Efficacy and Safety of Tramadol Once a Day 100, 200 and 300 mg Versus Placebo for the Treatment of Pain Due to Osteoarthritis of the Knee (With 7-Day Follow-up)	Included
	NCT00833794<br>2004-10/2006-01	A Two-Arm Study Comparing the Analgesic Efficacy and Safety of Tramadol HCl Once-a-Day Versus Placebo for the Treatment of Pain Due to Osteoarthritis	Included
	NCT00421928<br>2007-01/2008-12	Tapentadol (CG5503)	Included
	NCT00486811<br>2007-06/2008-07	A Study to Evaluate the Efficacy and Safety of CG5503 Prolonged Release (PR) in Subjects With Moderate to Severe Chronic Pain Due to Osteoarthritis of the Knee	Included
	NCT00487435<br>2007-06/2009-06	An Open-label Extension Study With Flexible Dosing of Extended-release (ER) Tapentadol (CG5503) to Treat Patients With Moderate to Severe Chronic Pain	Included
	NCT00531427<br>2007-09/2009-04	Buprenorphine Transdermal System (BTDS) in Subjects w/Mod-sev Osteoarthritis (OA) Chronic Pain of Knee	Included
	NCT00983073<br>2009-09/2010-09	Evaluation of Effectiveness and Tolerability of Tapentadol Hydrochloride in Subjects With Severe Pain Due to Osteoarthritis Taking Either WHO Step I or Step II Analgesics or no Regular Analgesic	Included
	NCT01124604<br>2010-04/2010-12	An Efficacy and Safety Study for Tapentadol Extended Release (JNS024ER) in Chronic Pain Participants	Included
	NCT01755546<br>2012-12/2014-11	Long-term Open-Label Safety Study to Evaluate EN3409	Included

	NCT00312221<br>2004-04/2005-08	Safety and Efficacy of Buprenorphine Transdermal System (BTDS) in Subjects With Moderate to Severe Osteoarthritis Pain	No control/placebo comparison
	NCT00420992<br>2006-12/2007-11	A Study of Embeda (Kadian NT, ALO-01) in Subjects With Pain Due to Osteoarthritis of the Hip or Knee	No relevant animal study identified
	NCT00631319<br>2008-02/2009-06	A Double-blind Study of Controlled Release OROS Hydromorphone Compared to Placebo in Patients With Chronic Osteoarthritis (OA) Pain	No data analysis reported
	NCT00736853<br>2008-06-2009-01	An Efficacy and Safety Study of Acetaminophen Plus Tramadol Hydrochloride (JNS013) in Participants With Chronic Pain	No relevant animal study identified
	NCT01008618<br>2009-01/2010-02	A Confirmatory Study of Fentanyl in Participants With Osteoarthritis or Low Back Pain	No relevant animal study identified
	NCT00979953<br>2009-10/2010-06	Efficacy and Safety Study Evaluating ADL5859 and ADL5747 in Participants With Pain Due to Osteoarthritis of the Knee	No data analysis reported
	NCT01240863<br>2010-11/2011-08	Study to Evaluate the Efficacy and Safety of Hydrocodone Bitartrate Extended-Release Tablets (CEP-33237) for Relief of Moderate to Severe Pain in Patients With Osteoarthritis or Low Back Pain Who Require Opioid Treatment for an Extended Period of Time	No relevant animal study identified
	NCT01298765<br>2011-03/2012-08	Longterm Safety Study of BEMA Buprenorphine in Subjects With Chronic Pain	No data analysis reported
	NCT01451385<br>2011-09/2012-06	Study of COV795 in Subjects With Osteoarthritis or Chronic Low Back Pain	No data analysis reported
	NCT01619839<br>2012-06/2013-09	A Phase 2 Study To Assess The Efficacy, Tolerability, And Safety OF NKTR-181 In Subjects With Chronic OA Knee Pain	No data analysis reported
	NCT02944448<br>2016-09/2017-05	A Study Evaluating Pain Relief and Safety of Orally Administered CR845 in Patients With Osteoarthritis of Hip or Knee	No data analysis reported
	NCT03008590<br>2018-05/2019-12	Low Dose Naltrexone for Chronic Pain From Arthritis	No relevant animal study identified
**Anti con**	NCT00408421<br>2006-11/2007-10	Duloxetine Versus Placebo for Osteoarthritis Knee Pain	Included

Supplementary table 1

	NCT00433290<br>2007-02/2008-05	Duloxetine vs. Placebo in the Treatment of Osteoarthritis Knee Pain	Included
	NCT00485472<br>2007-03/2008-01	Trial to Assess Efficacy and Safety of Lacosamide in Subjects With Osteoarthritis of the Knee	Included
	NCT00945945<br>2009-07/2010-05	A Study of Duloxetine in Patients With Osteoarthritis Knee Pain	Included
	NCT01931475<br>2013-09/2015-06	A Study of Duloxetine in Participants With Chronic Pain Due to Osteoarthritis in China	Included
	NCT02248480<br>2014-10/2015-06	A Study of Duloxetine (LY248686) in Participants With Chronic Osteoarthritis and Knee Pain in Japan	Included
	NCT02335346<br>2015-01/2016-03	An Extension Study of Duloxetine in Osteoarthritis and Knee Pain (Extension of F1J-JE-HMGX, NCT02248480)	Included
**TRPV1 modulator**	NCT03429049<br>2018-01/2019-11	A Phase 3 Efficacy and Safety Study of Intra-articular CNTX-4975-05 (Capsaicin) vs Placebo in Subjects With OA Knee Pain	Included
	NCT03528369<br>2018-05/2018-12	A Phase 2 Clinical Trial Examining the Effects on Osteoarthritic Knee Pain of CGS-200-1, CGS-200-5 and Vehicle Control	Included
	NCT03660943<br>2018-09/2020-06	A Clinical Study to Test Efficacy and Safety of Repeat Doses of CNTX-4975-05 in Patients With Osteoarthritis Knee Pain	Included
	NCT00878501<br>2009-03/2009-07	Study to Evaluate the Efficacy, Safety, Tolerability and Pharmacokinetics of AZD1386 in Patients With Osteoarthritis (OA) of the Knee	No data analysis reported
**Biological therapies**	NCT00399490<br>2006-09/2008-02	Open-label Extension Study Of RN624	Included
	NCT00669409<br>2008-06/2009-12	A Phase 1/2A, Single Dose Study Of PF-04383119 In Japanese Patients With Moderate To Severe Pain From Osteoarthritis Of The Knee	Included
	NCT00733902<br>2008-09/2010-01	Tanezumab in Osteoarthritis of the Knee	Included
	NCT00744471	Tanezumab in Osteoarthritis Of The Hip	Included

Supplementary table 1

	2008-11/2010-08		
	NCT00830063<br>2009-05/2010-08	Tanezumab In Osteoarthritis Of The Knee	Included
	NCT00863304<br>2009-06/2010-08	Tanezumab in Osteoarthritis of the Hip or Knee	Included
	NCT00985621<br>2009-10/2011-02	Tanezumab In Osteoarthritis Of The Hip Or Knee	Included
	NCT02192190<br>2014-07/2015-06	A Study of LY2951742 in Participants With Mild to Moderate Osteoarthritis Knee Pain	Included
	NCT02447276<br>2015-05/2016-11	Study of REGN475 in Patients With Pain Due to Osteoarthritis of the Knee or Hip	Included
	NCT02528188<br>2015-07/2019-02	Long Term Safety and Efficacy Study of Tanezumab in Subjects With Osteoarthritis of the Hip or Knee	Included
	NCT02683239<br>2016-02/2021-06	Long-Term Safety and Efficacy Study of Fasinuramab in Patients With Pain Due to Osteoarthritis (OA) of the Knee or Hip	Included
	NCT02709486<br>2016-03/2018-11	Study of the Analgesic Efficacy and Safety of Subcutaneous Tanezumab in Subjects With Osteoarthritis of the Hip or Knee.	Included
	NCT03161093<br>2017-08/2021-08	A Study to Determine the Safety and the Efficacy of Fasinuramab Compared to Placebo and Naproxen for Treatment of Adults With Pain From Osteoarthritis of the Knee or Hip	Included
	NCT03285646<br>2017-10/2019-05	Evaluate the Efficacy and Safety of Fasinuramab in Patients With Moderate-to-Severe Chronic Low Back Pain and Osteoarthritis of the Hip or Knee	Included
	NCT03304379<br>2017-10/2020-11	Study to Determine the Safety and the Efficacy of Fasunumab Compared to Placebo and Nonsteroidal Anti-inflammatory Drugs (NSAIDs) for Treatment of Adults With Pain From Osteoarthritis of the Knee or Hip	Included
	NCT03956550<br>2019-05/2020-10	A Study to Examine the Efficacy and Safety of REGN5069 in Patients With Pain Due to Osteoarthritis of the Knee	Included
	NCT00809354<br>2009-02/2011-01	Long-Term Analgesic Efficacy And Safety Of Tanezumab Alone Or In Combination With Non-Steroidal Anti-Inflammatory Drugs (NSAIDs) Versus NSAIDs Alone In Patients With Osteoarthritis Of The Knee Or Hip	No control/placebo comparison

Supplementary table 1

	NCT00864097 2009-08/2010-11	Analgesic Efficacy And Safety of Tanezumab Added On To Diclofenac SR In Patients With Osteoarthritis Of The Knee Or Hip	No control/placebo comparison
	NCT00994890 2009-11/2011-03	A Long Term Study of the Safety of Tanezumab When Administered By Subcutaneous Injections	No control/placebo comparison
	NCT02683785 2016-03/2017-11	A Study to Investigate the Efficacy and Safety of GSK3196165 in Inflammatory Hand Osteoarthritis	No relevant animal study identified
	NCT02674386 2016-08/2019-07	Long-term Observational Study of Subjects From Tanezumab Studies Who Undergo a Total Knee, Hip or Shoulder Replacement	No relevant animal study identified
	NCT03245008 2017-09/2021-03	Efficacy and Safety of MT-5547 in Patients With Osteoarthritis Accompanied by Moderate to Severe Pain	No relevant animal study identified
	NCT04456686 2020-07/2021-08	Chronic Pain Master Protocol (CPMP): A Study of LY3016859 in Participants With Osteoarthritis	No relevant animal study identified
	NCT04675034 2020-12/2023-08	A Study of the Efficacy and Safety of MEDI7352 in Participants With Painful Osteoarthritis of the Knee	No relevant animal study identified
**Other small molecules**	NCT00589979 2007-03/2008-10	Efficacy and Safety of the Lidoderm Patch Applied to Patients With Osteoarthritis of the Knee	Included
	NCT00589979 2008-06/2008-10	Efficacy and Safety of the Lidoderm Patch Applied to Patients With Osteoarthritis of the Knee	Included
	NCT01091116 2010-03/2011-03	A Locally Injected Bradykinin Antagonist for Treatment of Osteoarthritis	Included
	NCT02205814 2014-04/2015-01	Fasitibant Intra-articular Injection in Patients With Symptomatic Osteoarthritis of the Knee	Included
	NCT02536833 2015-09/2017-04	A Study Evaluating the Safety, Tolerability, and Efficacy of SM04690 Injected in the Target Knee Joint of Moderately to Severely Symptomatic Osteoarthritis Subjects	Included
	NCT02951026 2016-09	Evaluation of Safety and Efficacy of a Single Lorecivivint Injection in Patients with Knee Osteoarthritis: A Multicenter, Observational Extension Trial	Included
	NCT03122860	A Study Evaluating the Safety and Efficacy of SM04690 for the Treatment of Moderately to Severely Symptomatic Knee Osteoarthritis	Included

Supplementary table 1

	2017-04/2018-04		
	NCT03928184<br>2019-05/2021-08	Patient-Reported and Radiographic Outcomes in Evaluating Lorecivivint (SM04690) for the Treatment of Knee Osteoarthritis (STRIDES-Xray)	Included
	NCT04129944<br>2019-10/2020-08	A Study to Assess the Safety and Efficacy of a Single Dose of UBX0101 in Patients With Osteoarthritis of the Knee	Included
	NCT04385303<br>2020-05/2021-09	Patient-Reported Outcomes in Evaluating Lorecivivint (SM04690) for Moderate to Severe Knee Osteoarthritis (STRIDES-1)	Included
	NCT00041756<br>2002-07/2004-02	Efficacy and Safety of PG-530742 in the Treatment of Mild to Moderate Knee Osteoarthritis	No relevant animal study identified
	NCT00486434<br>2007-05/2010-09	Efficacy and Safety of Oral Salmon Calcitonin in Patients With Knee Osteoarthritis	No relevant animal study identified
	NCT00704847<br>2008-06/2011-06	Efficacy and Safety of Oral Salmon Calcitonin in Patients With Knee Osteoarthritis (OA 2 Study)	No relevant animal study identified
	NCT00790790<br>2008-11/2010-01	A Study in the Treatment of Osteoarthritis Knee Pain	No relevant animal study identified
	NCT01319929<br>2011-03/2011-09	A Study of LY2828360 in Patients With Osteoarthritic Knee Pain	No relevant animal study identified
	NCT01554579<br>2012-03/2013-11	A Four-Week Multicenter Study Evaluating the Safety and Efficacy of Gefapixant (AF-219/MK-7264) in Subjects With Osteoarthritis of the Knee	No data analysis reported
	NCT01654302<br>2013-09/2015-02	Efficacy and Safety of Synera in Osteoarthritis Pain	No data analysis reported
	NCT02068599<br>2014-04/2015-05	A Study to Evaluate the Safety and Efficacy of Topically Applied TV-45070 (Ointment) in Participants With Primary Osteoarthritis (OA) Affecting a Single Knee	No relevant animal study identified
	NCT02705625<br>2016-01/2017-05	A Study to Evaluate the Efficacy, Safety and Tolerability of MIV-711 in Osteoarthritis Patients	No relevant animal study identified
	NCT03028870<br>2017-03/2017-11	A Study to Evaluate the Analgesic Efficacy and Safety of V120083 in Subjects With Osteoarthritis (OA) of the Knee	No data analysis reported

Supplementary table 1

	NCT04627038<br>2020-11/2021-10	Chronic Pain Master Protocol (CPMP): A Study of LY3556050 in Participants With Osteoarthritis	No relevant animal study identified
	NCT04506463<br>2020-12/2022-08	Efficacy and Safety of MM-II for Treatment of Knee Pain in Subjects With Symptomatic Knee Osteoarthritis	No relevant animal study identified
	NCT05008835<br>2021-07/2022-11	Evaluate the Efficacy and Safety of ACP-044 in Subjects With Pain Associated With Osteoarthritis of the Knee	No data analysis reported
	NCT05080660<br>2021-10/2022-06	Chronic Pain Master Protocol (CPMP): A Study of LY3526318 in Participants With Osteoarthritis	No relevant animal study identified
	NCT05139030<br>2022-01/2022-07	Phase 3 Adductor Canal Block With EXPAREL in Subjects Undergoing Primary Unilateral Total Knee Arthroplasty	No control/placebo comparison
	NCT05620563<br>2022-11/2023-06	A Chronic Pain Master Protocol (CPMP): A Study of LY3857210 In Participants With Osteoarthritis Pain	No relevant animal study identified

Supplementary Table 2. **Participant characteristics in the selected clinical trials.** Data include the ClinicalTrials.gov identifier (NCT number), number of participants, age, percentage of female participants, body mass index (BMI), baseline pain level, and duration of pain symptoms. BMI and pain measures were extracted from study reports or inclusion criteria when available. *NA* indicates information not available. Abbreviations: VAS, Visual Analog Scale; WOMAC, Western Ontario and McMaster Universities Osteoarthritis Index; ACR, American College of Rheumatology; NRS, Numeric Rating Scale; BPI, Brief Pain Inventory; PGADS, Patient Global Assessment of Disease Status.

	NCT ID / Number of participants	Age (mean±SD)	Female (%)	BMI (mean±SD)	Pain at baseline	Pain duration
NSAID	NCT00092768<br>417	62±10	66	NA	WOMAC pain: 67±16	>6 months
	NCT00092781<br>419	62±9	65	NA	WOMAC pain: 67±16	>6 months
	NCT01461369<br>305	62±9	67	31.2±5.16	WOMAC: 72±16	NA
	NCT01554163<br>239	62±9	90	NA	[VAS pain score >800 mm (range: 0-100 mm)]	>6 months
	NCT01787188<br>402	61±9	66	30.9±5.01	WOMAC (range: 0-96): 69±16	NA
	NCT01980940<br>70	61±8	69	NA	NA	>6 months
	NCT02311881<br>770	61±8	63	NA	Moderate to moderately-severe self-reported pain	>3 months

	NCT03209362 80	60±8	51	30.63±4.44	NA	NA
	NCT05007808 210	65±8	60	NA	NRS (range: 0-10) : 7.6±1.35	>6 months
	NCT00664560 614	62±8	64	NA	[ACR functional class rating of I, II or III]	>6 months
	NCT00665431 610	62±8	64	NA	[ACR functional class rating of I, II or III]	>6 months
	NCT01147458 190	60±8	58	[≤39 kg/m²]	WOMAC Pain (range:0-20): 11.17 ± 2.67	NA
	NCT01119898 259	61±8	67	NA	Pain intensity when walking on a flat surface Moderate-sever pain	NA
	NCT01341405 125	61±8	90	NA	[WOMAC pain (range: 0-10): 4-8]	>3 months
	NCT01765296 362	62±8	85	NA	[WOMAC pain (range: 0-10): 4-8]	>3 months
	NCT01799213 490	58±11	11	NA	WOMAC pain (range: 0-20): 5.6±3.8	>3 months
	NCT01967550 304	65±7	65	NA	NA	>6 months
	NCT02067611	NA	NA	[18.5-39.9 kg/m²]	[VAS (range: 0-100 mm): 40-90 mm]	>6 months

Supplementary table 2

	216					
	NCT02121002<br>1175	NA	64	NA	[VAS (range: 0-100 mm)≥ 20 mm]	NA
	NCT02311881<br>770	61±8	63	NA	[Moderate to moderately-severe self-reported pain]	>3 months
	NCT02913521<br>786	57±11	74	NA	[WOMAC pain (range: 0-20): 9]	>6 months
	NCT02596451<br>1143	56±11	64	NA	[WOMAC pain (range: 0-20): 9]	>6 months
	NCT03172780<br>1220	53±10	69	NA	WOMAC pain (range: 0-20): 12.4±2.12	NA
	NCT03277066<br>283	58±9	26	[≤40 kg/m²]	NA	>6 months
	NCT03691844<br>44	NA	67	[≤45 kg/m²]	NRS (range: 0-50) : 27.5 ±5.41	>3 weeks
**Corticosteroid**	NCT02116972<br>310	59±8	57	[≤40 kg/m²]	NA	>6 months
	NCT02357459<br>486	62±10	61	[≤40 kg/m²]	NA	>6 months
	NCT00521989<br>279	59±9	63	NA	[WOMAC pain (range: 0-100): 30-80 mm]	>6 months

Supplementary table 2

	NCT01487161228	62	53	[≤40 kg/m²]	[NRS (range: 0-10): 5-9]	NA
	NCT024685836	42	20	NA	NA	NA
	NCT02688400380	64±7	75	NA	[VAS while walking on a flat surface (range: 0-100 mm) ≥ 40 mm]	NA
	NCT0300587375	64±9	50	NA	VAS (range: 0-10): 6.62 ± 1.14	>6 months
	NCT0319190357	58±9	68	[≤40 kg/m²]	WOMAC pain (range: 0-100 mm): 63.4±11.2	>6 months
	NCT0339003652	58±9	69	NA	WOMAC pain (range: 0-100 mm): 63.7 +/- 11.2	NA
	NCT0379301068	61±9	60	30.91±5.70	[WOMAC pain (range:0-10)]	>3 months
	NCT0416009110	61±11	20	29.45±2.80	[NRS (range: 0-10)≥3]	>1 month
	NCT0412356150	61±9	59	31.35±4.83	[NRS (range: 0-10)≥4]	NA
	NCT0423131823	59±9	61	[≤40 kg/m²]	[WOMAC pain (range: 0-100 mm): 50-90 mm]	>6 months

Supplementary table 2

	NCT04120402 318	64±9	58	[≤40 kg/m²]	NA	>6 months
**Opioid**	NCT00950651 860	61±9	83	[≤35 kg/m²]	[WOMAC pain subscale (range: 0-500)≥ 150 mm]	>3 months
	NCT00832416 565	60±9	60	[≤38 kg/m²]	[WOMAC pain subscale (range: 0-500)≥ 150 mm]	NA
	NCT00852917 552	61±9	62	[≤38 kg/m²]	[WOMAC pain subscale (range: 0-500)≥ 150 mm]	NA
	NCT00833794 646	62±9	63	[≤37 kg/m²]	[NRS (range: 0-10)≥4]	NA
	NCT00421928 1023	58±10	60	NA	[Pain intensity the last 3 days (range: 0-11) ≥ 5]	>3 months
	NCT00486811 987	62±9	72	NA	[Pain intensity the last 3 days (range: 0-11) ≥ 5]	>3 months
	NCT00487435 1154	54±11	58	NA	NA	NA
	NCT00531427 567	59±10	63	NA	NA	>12 months
	NCT00983073 224	67±11	68	NA	NA	>3 months

	NCT01124604 91	65±15	68	NA	[Pain intensity the last 3 days (range: 0-11) ≥ 5]	>3 months
	NCT01755546 224	52±12	56	32.10±7.20	Moderate to severe pain	>3 months
	NCT00313846 326	61±10	57	NA	Moderate to severe pain	>12 months
	NCT00312221 418	58±10	74	NA	NA	>12 months
	NCT00420992 547	54±12	58	NA	NA	>3 months
	NCT00980798 288	65±10	72	NA	[BPI (range: 0-10) ≥ 5]	>3 months
	NCT00631319 343	56±10	54	NA	NA	>6 months
	NCT00736853 321	57±18	61	NA	NA	>3 months
	NCT01008618 218	67±13	67	NA	[VAS≥50 mm]	>3 months
	NCT00982280 63	65±10	59	NA	[NRS (range: 0-10)≥ 5]	>3 months

Supplementary table 2

	NCT00979953 408	57±8	59	NA	[NRS (range: 0-10)≥ 4]	>6 months
	NCT01240863 294	53±11	60	32.90±7.74	[NRS (range: 0-10)≥ 5]	>3 months
	NCT01298765 302	56±13	61	32.23±6.85	NRS (range: 0-10): 3.3 ±1.94	>3 months
	NCT01451385 376	52±14	59	NA	NRS (range: 0-10): 6.7±1.64	>3 months
	NCT01619839 213	60±10	60	[18-39 kg/m²]	NA	NA
	NCT02944448 761	61±10	63	[≤40 kg/m²]	[NRS(range: 0-10)≥ 5]	NA
	NCT03008590 23	63±10	17	34±7.3	BPI (range:0-10): 5.9±1.2	NA
**Anticonvulsants and antidepressants**	NCT00408421 231	62±9	65	[≤40 kg/m²]	BPI (range:0-10): 6.20±1.56	Pain onset: 9.17±8.47 years
	NCT00433290 256	63±9	77	29.55±4.58	BPI (range:0-10): 6.11±1.33	Pain onset: 7.44±7.15 years
	NCT00485472 149	62±7	75	NA	NA	>6 months
	NCT00945945	64±10	68	[≤40 kg/m²]	[BPI (range: 0-10) ≥ 4]	>3 months

	424					
	NCT01931475 407	61±8	76	[≤40 kg/m²]	[BPI (range: 0-10) ≥ 4]	>3 months
	NCT02248480 353	66±8	78	NA	[BPI (range: 0-10) ≥ 4]	>3 months
	NCT02335346 93	66±8	72	NA	[BPI (range: 0-10) ≥ 4]	>3 months
	NCT01018680 524	61±9	57	[≤40 kg/m²]	BPI (range: 0-10): 6.16±1.54	>3 months Pain onset: 9.49±8.90 years
**TRPV1 modulator**	NCT03429049 325	62±9	63	31.77±5.73	WOMAC pain (range: 0-50): 31.45±7.79	>6 months
	NCT03528369 122	60	63	NA	WOMAC pain (range: 0-500): 351.5 (254 to 490)	>3 months
	NCT03660943 324	63±9	61	32.38±5.62	WOMAC (range: 0-96): 31.5±7.28	>6 months
	NCT00878501 241	62	69	NA	NA	NA
**Biological therapies**	NCT00399490 287	59±8	61	NA	NA	NA
	NCT00669409 83	NA	69	NA	NA	NA

	NCT00733902 690	62±10	61	[≤39 kg/m²]	NA	NA
	NCT00744471 621	NA	62	[≤39 kg/m²]	NA	NA
	NCT00830063 828	61±10	60	[≤39 kg/m²]	NA	NA
	NCT00863304 849	NA	64	NA	NA	NA
	NCT00985621 614	57±10	63	[≤39 kg/m²]	NA	NA
	NCT02192190 268	58±9	62	NA	NA	NA
	NCT02447276 342	61±8	65	31.12±4.9	[WOMAC pain >4]	NA
	NCT02528188 3021	61±9	65	NA	[WOMAC pain (range: 0-20): ≥5]	NA
	NCT02683239 5331	64±9	73	NA	[WOMAC pain (range: 0-20): ≥4]	>3 months
	NCT02709486 849	65±8	69	NA	[WOMAC pain (range: 0-20): ≥5]	NA
	NCT03161093	62.9 (8.85)	5	NA	[Moderate to severe pain]	>3 months

	3307					
	NCT03285646 63	59±10	62	NA	NRS (range: 0-10): 6.73±1.40	NA
	NCT03304379 1650	62±9	70	NA	WOMAC pain (range: 0-20): 6.42±1.34	>3 months
	NCT03956550 259	64±9	80	[≤39 kg/m²]	WOMAC pain (range: 0-20): 6.36±1.18	NA
	NCT00809354 2700	NA	71	[≤39 kg/m²]	NA	NA
	NCT00864097 607	NA	78	[≤39 kg/m²]	NA	NA
	NCT00994890 679	NA	69	[≤39 kg/m²]	NA	NA
	NCT02683785 44	59±7	91	NA	[NRS (range: 0-10)≥ 5]	NA
	NCT02674386 150	65±8	59	NA	NA	NA
	NCT03245008 62	67±9	79	26.8±4.3	WOMAC pain (range: 0-20): 6.02 ± 1.35	NA
	NCT04456686 117	63±9	77	[≤39 kg/m²]	NRS (range: 0-10): 5.61 ±1.52	>3 months

Supplementary table 2

	NCT04675034345	64±9	67	NA	NA	NA
Other small molecules	NCT0058997993	61±10	65	[≤40 kg/m²]	NA	NA
	NCT01091116423	66±9	60	NA	WOMAC pain (range: 0-500 mm): 286±74	6 months<br>Pain onset: 8 years
	NCT02205814431	64±8	61	26.9±2.50	WOMAC pain (range: 0-500 mm): 280.8±39.61	NA
	NCT02536833455	60±9	59	[≤40 kg/m²]	[VAS (range: 0-100 mm): 30-80 mm]	>6 months
	NCT02951026703	61±9	61	29.05±4.3	[WOMAC pain (range: 0-100) ≥ 60]	>6 months
	NCT03122860694	59±9	58	28.97±4.01	NA	>6 months
	NCT03928184501	60±8	66	[≤40 kg/m²]	[NRS (range: 0-10)≥4]<br>[WOMAC pain (range: 0-50 mm): 20-40]	
	NCT04129944183	64	36	[≤40 kg/m²]	[NRS (range: 0-10): 4-9]	NA
	NCT04385303470	61±8	60	[≤35 kg/m²]	NA	>6 months
	NCT00041756	63±0.4	71	NA	NA	NA

Supplementary table 2

	395					
	NCT00486434<br>1176	64±7	68	NA	NA	NA
	NCT00704847<br>1030	64±7	61	NA	NA	NA
	NCT00790790<br>147	59±7	69	[≤40 kg/m²]	[Pain score (range: 0-10): 4-8]	>3 months
	NCT01319929<br>39	63±7	36	[19-35 kg/m²]	[Pain score (range: 0-10): 4-8]	>3 months
	NCT01554579<br>171	NA	64	NA	[NRS (range: 0-10): 5-9]	NA
	NCT01654302<br>38	58±8	61	NA	Knee pain after exercise intervention (range: 1-10): 6.08±1.33	>1 months
	NCT02068599<br>38	60±9	59	28.17±3.11	NA	NA
	NCT02705625<br>240	62±7	77	NA	[NRS (range: 0-10): ≥4]	NA
	NCT03028870<br>291	60±9	63	NA	[NRS (range: 0-10): 4-9]	>6 months
	NCT04627038<br>202	63±9	57	[≤40 kg/m²]	[VAS (range: 0-100 mm): 40-95 mm]	>3 months

	NCT04506463 369	NA	65	NA	NA	>6 months
	NCT05008835 61	57±5	67	[≤39 kg/m²]	NRS (range: 0-10): 5.7±1.55	NA
	NCT05080660 160	64±9	63	[≤40 kg/m²]	NRS (range: 0-10): 5.65±1.50	>3 months
	NCT05139030 166	62±8	49	32.07±4.90	NRS (range: 0-10): 5.2±2.27	NA
	NCT05620563 147	63±9	87	[≤40 kg/m²]	NRS (range: 0-10): 5.57±1.64	>3 months

Supplementary table 2
